# Reactions of the Lipid Hydroperoxides With Aminic Antioxidants: The Influence of Stereoelectronic and Resonance Effects on Hydrogen Atom Transfer

**DOI:** 10.3389/fchem.2019.00850

**Published:** 2019-12-17

**Authors:** Yu-Zhen Li, Xiao-Lu Zhou, Bao-Qi Huo, De-Zhan Chen, Zhao-Hua Liu, Xie-Huang Sheng

**Affiliations:** ^1^College of Chemistry, Chemical Engineering and Materials Science, Collaborative Innovation Center of Functionalized Probes for Chemical Imaging in Universities of Shandong, Key Laboratory of Molecular and Nano Probes, Ministry of Education, Shandong Provincial Key Laboratory of Clean Production of Fine Chemicals, Shandong Normal University, Jinan, China; ^2^Key Laboratory of Systems Bioengineering, Ministry of Education, Department of Pharmaceutical Engineering, School of Chemical Engineering and Technology, Tianjin University, Tianjin, China; ^3^Center for New Drug Evaluation, School of Pharmaceutical Sciences of Shandong University, Jinan, China

**Keywords:** aminic RTAs, ferroptosis, stereoelectronic, synergetic characteristics, resonance factors

## Abstract

Aminic radical-trapping antioxidants (RTAs), as one of the most important antioxidants, have not received sufficient attention yet. But, an increasing number of aminic RTAs have been identified as ferroptosis inhibitors in recent years, which can potentially mediate many pathological states including inflammation, cancer, neurodegenerative disease, as well as ocular and kidney degeneration. This highlights the importance of aminic RTAs in the field of medicine. Herein, we systematically explored the radical scavenging mechanism of aminic RTAs with a quantum chemical method, particularly emphasizing the role of stereoelectronic factors and resonance factors on the transfer of H-atom and the stability to one-electron oxidation. These theoretical results elucidate the diversity of free radical scavenging mechanisms for aminic RTAs, and has significant implications for the rational design of new aminic RTAs.

**Graphical Abstract:**
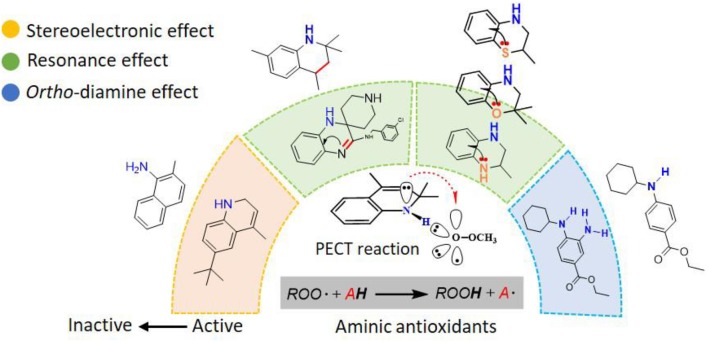
Reactions of the Lipid Hydroperoxides with aminic antioxidants: The Influence of Stereoelectronic and Resonance Effects on Hydrogen Atom Transfer.

## Introduction

Lipid peroxidation, the autoxidation of biological lipids, is an oxidative damage that affects cellular membranes, lipoproteins, and other molecules which contain lipids in conditions with oxidative stress (Yin et al., 2011). It is a well-defined mechanism of cellular damage in both animals and plants that occurs *in vivo* during aging and in certain disease states (Massey and Nicolaou, 2013). For example, the pathogenesis of cancer, neurodegeneration, and atherosclerosis have been linked to lipid peroxidation (Poon and Pratt, 2018). Recently, a novel regulated non-apoptotic cell death termed ferroptosis (Dixon et al., 2012) was characterized, which can clearly explain the significant correlation between lipid peroxidation and the pathogenesis of neurodegenerative diseases (Stockwell et al., 2017), such as Parkinson's (Deas et al., 2016), Huntington's (Paul et al., 2014), and Alzheimer's diseases (Chen et al., 2015), traumatic (Zille et al., 2017), and hemorrhagic brain injury (Li et al., 2017). And the inhibition of membrane lipid autoxidation has been shown to help alleviate these diseases (Angeli et al., 2017).

**Figure 1:**
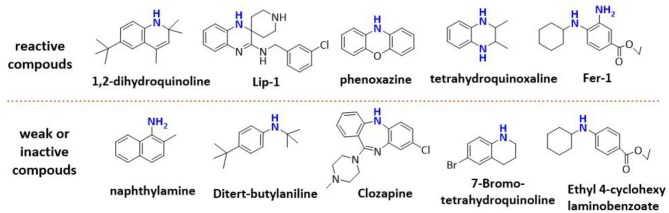
The representative compounds of aminic antioxidants.

Phenols have been studied extensively as radical trapping antioxidants (RTAs) and their structures have been optimized for use in a wide variety of contexts (Poon and Pratt, 2018). However, all the newly discovered ferroptosis inhibitors are belonging to aromatic amines acting as potent RTAs (Friedmann Angeli et al., 2014; Skouta et al., 2014; Shah et al., 2017; Sheng et al., 2018) (Figure 1; Figure S1). This might be concerned with the convenience of adjusting molecular lipophilicity for aminic RTAs to scavenge free radicals in cell membranes. Furthermore, amines offer greater structural variability owing to their trivalent central nitrogen atom compared with classic phenolic RTAs (Figure 1). As such, the reactivity -NH group can be in a ring or links two functional groups simultaneously. With the discovery of larger numbers of aminic RTAs, aminic compounds would become an exciting class of antioxidants for clinical use.

However, the antioxidant mechanism of some aminic RTAs is still confused. For instance, compared to phenoxazine, clozapine has the similar structure but presents a very weak antioxidant activity (Figure 1). Thus, in this work, we collect the recent reported aminic RTAs and systematically describe their lipid radical trapping mechanism by means of density functional theory (DFT) calculations as well as natural bond orbital (NBO) analysis. In addition, we also focus on the stereoelectronic factors of amine group account for the diversity of antioxidant function. These quantum-chemical details would allow us to uncover the structure basis for the antioxidant potency of aminic RTAs and provide invaluable models for design of novel antioxidants and ferroptosis inhibitors.

### The General Radical-Trapping Property of Aminic Antioxidants

At first, it should be noted that the majority of newly identified aminic RTAs are derived from ferroptosis inhibitors (Friedmann Angeli et al., 2014; Skouta et al., 2014; Shah et al., 2017; Sheng et al., 2018). Thus, their activity data are mainly obtained from the inhibition potency of ferroptosis not supported by the antioxidant activity test directly. Many studies have already demonstrated that the capacity of scavenging lipid free radicals was straightly correlated with the anti-ferroptotic cytoprotective activity (Dixon et al., 2012; Sheng et al., 2017, 2018; Zilka et al., 2017). In this work, we first present the relationship between the theoretical energy barriers of H-atom transferring from aminic RTAs to peroxyl radicals and anti-ferroptotic cytoprotective activities.

**Figure 2:**
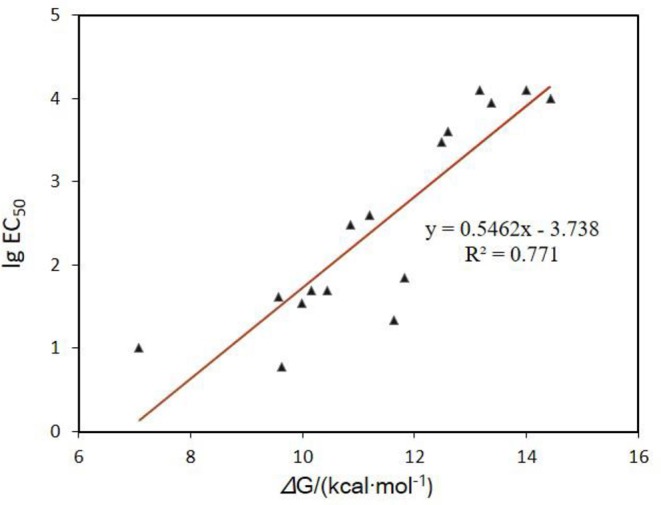
Correlation of the cytoprotective potency of RTAs against ferroptosis and the theoretical energy barriers of H-atom transferring from the arylamine moieties to methylperoxy radical (CH_3_OO•).

**Figure 3:**
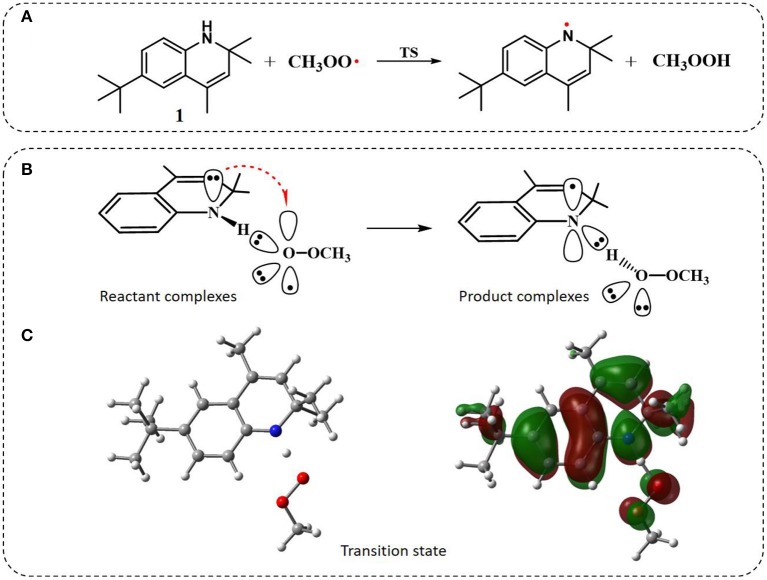
The general property of aminic RTAs in trapping free radical. **(A)** The chemical reaction formula between aminic RTAs (**1**) and CH_3_OO•. **(B)** The chart of proton-coupled electron transfer (PCET) reaction. **(C)** Optimized structure and HOMO orbitals of the transition state.

Aromatic amines are a well-known class of radical scavengers and chain-breaking antioxidants. Their reactivity potency are heavily dependent on the rapid transfer of a H-atom from the arylamine moieties to methylperoxy radical (CH_3_OO•) (Poon and Pratt, 2018) (Figure 3A). As shown in Figure 2, the activation energy barrier has a good linear relationship with the cytoprotective potency of RTAs against erastin-induced ferroptosis (Figure 2; Table S1). These compounds, which have lower energy barriers (e.g., **1**, Δ*G* = 11.86 kcal mol^−1^), exhibit higher inhibitory potency against ferroptosis (e.g., **1**, EC_50_ = 70 nM) (Shah et al., 2017). These data further substantiate the tight correlation between the antioxidant capacity of aminic RTAs and the anti-ferroptotic cytoprotective activity. Therefore, it is plausible to study the mechanism of aminic radical-trapping antioxidants by using the activity information of anti-ferroptotic effect.

Similarly to phenols, aminic RTAs transfer their aminic H-atom to peroxyl radicals via transition state (TS) in which a pre-reaction H-bonding will be formed between the -NH group of aminic RTAs and the O lone pair on the peroxyl radicals (DiLabio and Johnson, 2007; Hanthorn et al., 2012). As described in excellent works by Derek A. Pratt group (Hanthorn et al., 2012; Poon and Pratt, 2018), the mechanism of aminic RTAs with peroxyl radicals is a typical proton-coupled electron transfer (PCET) reaction, wherein the proton moves from the amine to the peroxyl radical via two nominally non-bonding orbitals while an electron moves from the π-HOMO of the amine to the π-LUMO of the peroxyl radical (Figure 3B) (Ingold and Pratt, 2014). This orbitals involved in the electron transfer are a combination of the partially bonding π-orbital involving the N atom in the TS structure and the N lone pair–ring π overlap (Figure 3C) (DiLabio and Johnson, 2007). Thus, the conjugation degree between N lone pair and the aromatic π-system can seriously determine the rate of electron transfer. The process of PCET happened in heteroatom/heteroatom mechanism is energetically more favorable than H exchange by the hydrogen atom transfer (HAT) mechanism (Mayer et al., 2002).

Additionally, the stability to one-electron oxidation is another determine factor for the potency of antioxidants (Farmer et al., 2017). Thus, we will elaborately discuss the types involved in reactivity to H-atom transfer and stability to one-electron oxidation of aminic RTAs in the following context.

### Stereoelectronic Factor Determine the Efficacy of Aminic RTAs

In naphthylamine (**2**), its lowest energy conformation is planar because this geometry allows for the good alignment of the p-type lone pair at nitrogen with the aromatic π-system (reactant, Table 1). The energy of ${\text{n}}_{\text{N}}\to \pi^{*}$ interaction between nitrogen and the aromatic π-system is 30.27 kcal mol^−1^. But, when the lipid radical (CH_3_OO•) closes to –NH_2_ group (TS, Table 1), the newly formed H-bonding induce the -NH group out of the plane of the naphthalene ring due to the steric repulsion effect. In such systems, the nitrogen lone pair can assume a misaligned orientation, with the considerable loss of the ${\text{n}}_{\text{N}}\to \pi^{*}$ interaction (15.43 kcal mol^−1^). Correspondingly, the loss of the ${\text{n}}_{\text{N}}\to \pi^{*}$ resonance can strongly confine the electron transferring form nitrogen to the orbital of radical and finally cause the whole reaction energetically unfavorable with the activation energy barrier of 14.43 kcal mol^−1^. It reveals why compounds containing an exocyclic amine generally do not have high reactivity toward peroxyl radicals.

**Table 1:** Stereoelectronic effect of intracyclic amine and exocyclic amine in trapping lipid radical.

**Compound no**.	**Reactant**	**TS**
**1** (active)	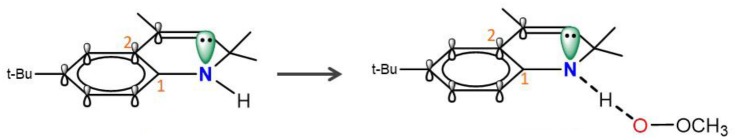	
	φ = 171.69°	φ = 171.94°
	n_N_ → π* = 39.05	n_N_ → π* = 27.72
		*Δ*G = 11.83 kcal·mol^−1^
**2** (inactive)	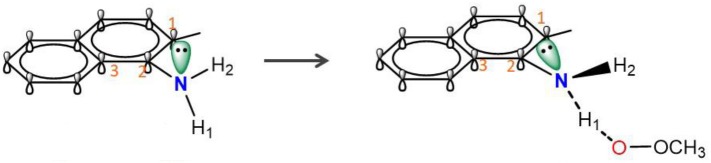	
	Φ = 11.56°	Φ = 45.97°
	n_N_ → π* = 30.27	n_N_ → π* = 15.43
		*Δ*G = 14.43 kcal·mol^−1^

*φ = H-N-C_1_-C_2_; φ = (H_1_-N-C_2_-C_1_) – (H_2_-N-C_2_-C_3_)*.

The rotatability and steric hindrance effect of exocyclic amine render the molecule of naphthylamine inactive. Interestingly, this problem can be overcome by confining the functional -NH group to a ring in which structural constraints for steric disturb the conjugation between lone pair at nitrogen and aromatic π-system, such as compound **1** (reactant, Table 1). For the antioxidant reaction of compound **1**, the methylperoxy radical attacks along the N-H direction and have no apparent steric repulsion with dihydroquinoline ring. In this case, -NH group endures almost no rotation and maintains a good alignment of the p-type lone pair at nitrogen with the aromatic π-system (TS, Table 1). The energy of ${\text{n}}_{\text{N}}\to \pi^{*}$ interaction between nitrogen and the aromatic π-system in TS state is 27.72 kcal mol^−1^. Thus, this strong conjugation between nitrogen and the aromatic π-system can greatly increase the PCET rate and lower the activation energy (Δ*G* = 11.83 kcal mol^−1^) for reaction with a peroxyl radical.

Phenoxazine (**3**) and clozapine (or olanzapine) are both tricyclic compounds but exhibit a significantly different antioxidant capacity. For phenoxazine, it adopts a planar conformation to allow the perfect alignment of the electron lone pair of nitrogen with the π-system, accompanied by the ${\text{n}}_{\text{N}}\to \pi^{*}$ interaction energy reaching up to 40.66 kcal mol^−1^ (reactant, Table 2). And the TS structure still maintains this good alignment (Table 2). Thus, the extended conjugated system significantly promotes PCET electron transfer and greatly increases the rate of H-atom transfer. Moreover, the structure of phenoxazine has an extensive π-system which is concomitant with an increase of spin delocalization of the aminyl radical. The remarkable balance in H-atom transfer kinetics and stability to one-electron oxidation leads to phenoxazine and its derivatives being considered to be the fastest RTAs reported to date, which undergo barrierless HAT reactions with peroxyl radicals (Δ*G* = 7.08 kcal mol^−1^, Table 2) (Poon and Pratt, 2018). *Derek A. Pratt* has even pointed out that phenoxazine is a privileged scaffold for RTAs development (Farmer et al., 2017).

**Table 2:** Stereoelectronic effect of planar tricyclic amine and non-planar tricyclic amine in trapping lipid radical.

**Compound no**.	**Reactant**	**TS**
**3** (active)	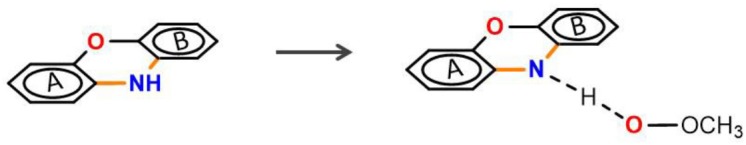	
	φ = 0.03°	φ = 18.29°
	n_N_ → π*_A_ = 40.66	n_N_ → π*_A_ = 20.71
	n_N_ → π*_B_ = 40.66	n_N_ → π*_B_ = 20.86
		*Δ*G = 7.08 kcal mol^−1^
**4** (inactive)	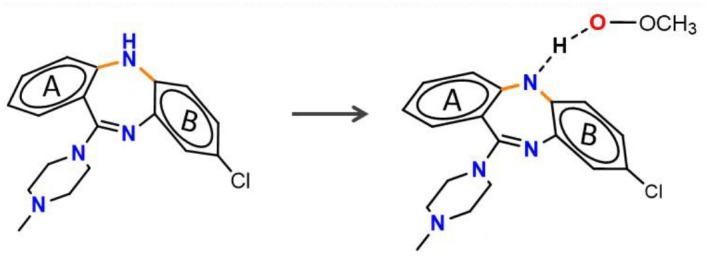	
	φ = 119°	φ = 149°
	n_N_ → π*_A_ = 18.14	n_N_ → π*_A_ = 17.50
	n_N_ → π*_B_ = 19.97	n_N_ → π*_B_ = 17.89
		*Δ*G = 13.99 kcal mol^−*1*^

*φ: the dihedral angle between ring A and ring B; all energies are in kcal mol^−1^*.

As for clozapine, relative to phenoxazine, it is not a planar structure with the dihedral angle between two phenyl rings of 119° and partial loss of the ${\text{n}}_{\text{N}}\to \pi^{*}$ interaction. While reacting with peroxyl radical, the tricyclic structure of clozapine becomes more planar (φ = 149°, Table 2). The better conjugation of nitrogen with the aromatic ring seems to increase the PCET rate. However, the remarkable change of dihedral angle (Δφ = 30°) tricyclic structure requires great energy (+6.93 kcal mol^−1^, Table S2) and ultimately makes the overall reactions unfavorable, with the activation energy barrier of 13.99 kcal mol^−1^. Thus, the conformation change is the dominant factor for the low potency of clozapine.

Above discussions highlight the importance of stereoelectronic effects for the antioxidant potency of aminic RTAs. This effect is crucial from the practical point of view because small differences lead to large inhibitory potency shifts and minimize the problems needed a careful consideration of the conjugation of reactive -NH groups with aromatic π-system. Collectively, these mechanisms suggest that planar, intracyclic amine compounds are a good choice for antioxidants.

### The Resonance Factor Determines the Reactivity of Aminic RTAs

Besides the fast H-atom transfer kinetics, stability to one-electron oxidation is another important factor for the reactivity of aminic RTAs (Farmer et al., 2017). In this section, we will discuss two distinct types of resonance overlap in determining the efficacy of aminic RTAs.

**Figure 4:**
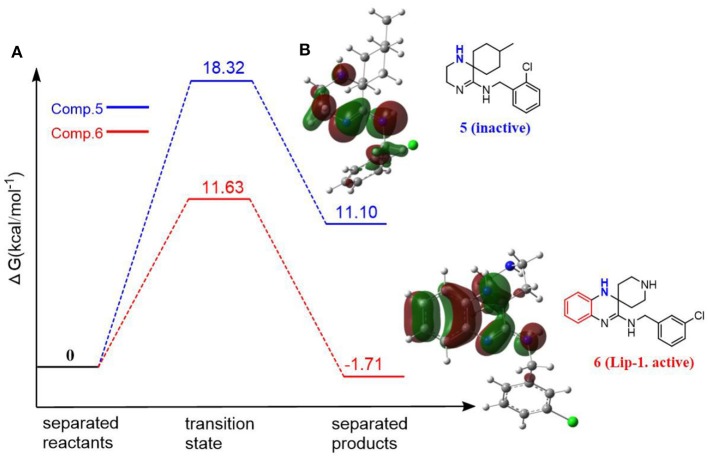
The resonance effect on the potency of aminic RTAs. **(A)** Potential energy profiles for CH_3_OO• with compound 5 and compound 6 (values are given in kcal·mol^−1^), **(B)** HOMO orbitals of compounds (molecular orbitals are rendered at an isovalue of 0.002).

As phenolic compounds, whose antioxidant activity is due primarily to resonance stabilization of the phenoxyl radical after oxidation (Zhou and Elias, 2013), the molecules containing highly conjugate structure is also the fundamental property for aminic RTAs. For example, extensive conjugation of the nitrogen p-lone pair with the dihydroquinoxaline have been found in compound **5** (liproxstatin-1, Lip-1) which can better stabilize the aminyl radical by resonance after reaction occurs (Sheng et al., 2017). In this case, the hydrogen abstraction from -NH bond in Lip-1 is expected to be favored with the calculated energy barrier of 11.63 kcal·mol^−1^, and the whole reaction is an exothermic process (1.71 kcal·mol^−1^, Figure 4). But for compound **6** (Friedmann Angeli et al., 2014), its structure lacks a benzene ring compared with compound **5**. Benzene is usually recognized as one of the most important conjugated structure for organic compounds. Eventually, a considerably restricted delocalization of unpaired electron spin in compound **6** product radical make parent structure extremely unfavorable in scavenging peroxyl radical with the reactive energy barrier up to 18.32 kcal·mol^−1^ (Figure 4).

### π-π Conjugation

Our next objective is to elucidate the types of resonance overlap for the reactivity of aminic RTAs with lipid peroxyl radical. As shown in Table 3, tetrahydroquinoline analogs (**7**, **8**, Table 3) have been observed extremely weak reactivity in trapping free radical, but the inhibitory potency increase sharply just adding a double bond in the molecules, such as dihydroquinoline analog (**1**) and dihydroquinoxaline (**6**, Figure 4) (Friedmann Angeli et al., 2014; Shah et al., 2017). In particular, the required energy barriers for tetrahydroquinoline (**7**, Δ*G* = 13.38 kcal mol^−1^; **8**, Δ*G* = 13.16 kcal mol^−1^) are significantly higher than that of dihydroquinoline (***1***, Δ*G* = 11.83 kcal mol^−1^). It is implied that the high potency of dihydroquinoline analog is due to the resonance stabilization enhanced by the conjugation between π electrons of the aromatic ring and the π double bond. In other word, the π-π conjugation composed by a π double bond with aromatic π system is the minimum degree of resonance for the reactivity of bicyclic aminic RTAs.

**Table 3:** The relationships between antioxidant activity (*ΔG*) and the extent of π-π conjugation.

**Compound no**.	**Structures**	**HOMO**	**Energy barrier (*Δ*G, kcal mol^**−1**^)**
**1** (active)	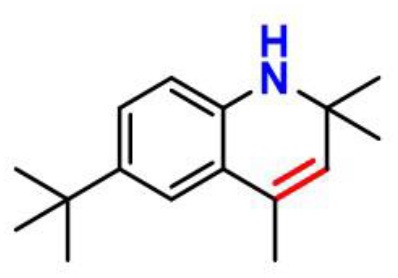	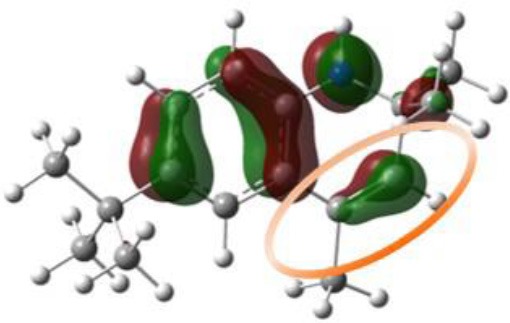	11.83
**7** (inactive)	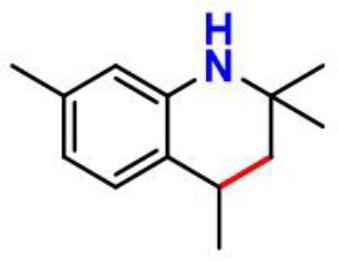	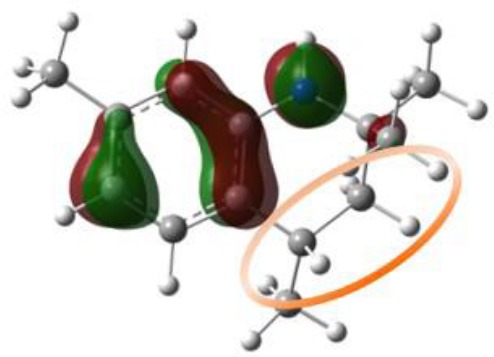	13.38
**8** (inactive)	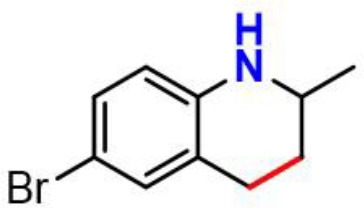	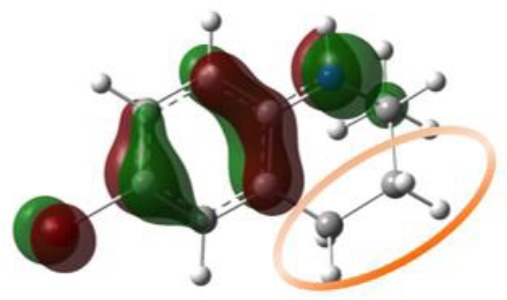	13.16

*Molecular orbitals are rendered at an isovalue of 0.002*.

### p-π Conjugation

In addition to π-π conjugation, p-π conjugation effect is another mainly conjugation effects. In recent, we have identified tetrahydroquinoxaline deviates as potent ferroptosis inhibitors which contain a typical p-π conjugation. Remarkably, as shown in Table 4, the potency of these compounds against lipid peroxyl radical is largely dependent on the degree of resonance overlap. The *p*-orbital of nitrogen in tetrahydroquinoxaline **11** is overlap best to the aromatic π-system with the ${\text{n}}_{\text{N}}\to \pi^{*}$ interaction energies [*E(2)* values] of 33.60 kcal mol^−1^. In this case, compounds **11** require the lowest energy barrier (9.97 kcal mol^−1^) in the process of trapping lipid peroxyl radical. However, the introduction of oxygen and sulfur of 2H-benzoxazine (**10**) and 2H-benzothiazine (**9**) significantly decrease the ${\text{n}}_{\text{N}}\to \pi^{*}$ interaction energies and weaken the resonance stabilization of the phenoxyl radical after reaction. Obviously, their inhibitory potency would decrease remarkably, with nearly 10-fold and 100-fold less potent than that of tetrahydroquinoxaline **11**. These results indicate that the strong p-π conjugation has played an important role in trapping free radical for bicyclic aminic RTAs.

**Table 4:** The relationships between antioxidant activity (*ΔG*) and the extent of *p-*π conjugation.

**Compound no**.	**Structure**	***p-π* conjugation (*E2* value, kcal mol^**−1**^)**	**Energy barrier (*Δ*G, kcal mol^**−1**^)**
**9** (low activity)	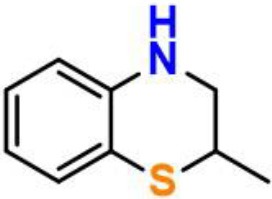	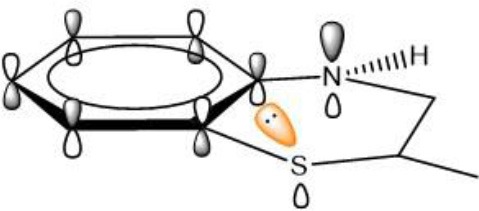	12.49
		${\text{n}}_{\text{N}}\to \pi^{*}$ = 18.34	
**10** (medium activity)	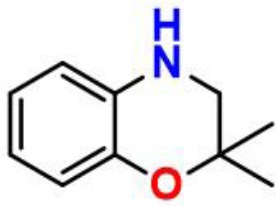	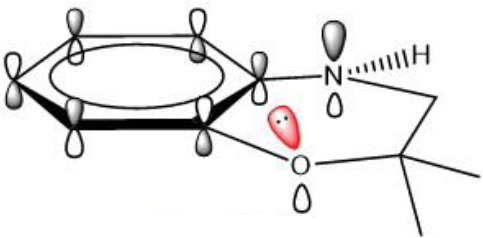	10.85
		${\text{n}}_{\text{N}}\to \pi^{*}$ = 28.15	
**11** (high activity)	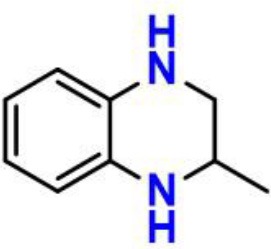	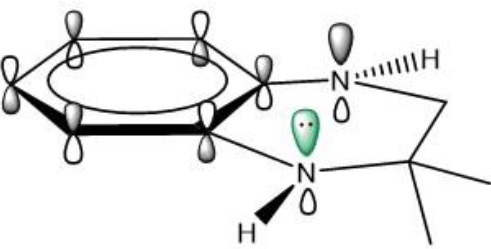	9.97
		${\text{n}}_{\text{N}}\to \pi^{*}$ = 33.60	

### The Synergetic Characteristics of *Ortho*-diamine RTAs

As discussed above, the inhibitory potency of the mono-aminic RTAs (**2, 13**) is extremely weak due to the misalignment of p-type lone pair at reactive nitrogen with the aromatic π-system in the TS state. But, the inhibitory reactivity would be reversed by the addition of -NH group at *ortho* site (Skouta et al., 2014). In compound **12**, two hydrogen atoms from *ortho*-diamines moiety can simultaneously interact with the methylperoxyl radical CH_3_OO• and form a near-planar seven-membered heterocyclic ring (Table 5) (Hammond, 1955; Sheng et al., 2018), which is favorable for unpaired electron delocalization through π-conjugation. This geometry helps to stabilize the transition state and substantially decrease the activation energy. The calculated barrier of compound **12** with methylperoxyl radical is 10.45 kcal mol^−1^, far less than that of compound **13** (18.80 kcal mol^−1^). It is consistent with the experiment result that compound **12** expresses full protection from erastin-induced ferroptosis while compound **13** does not show any activity at all (Sheng et al., 2018).

**Table 5:** The o-substituent affect the stability to one-electron oxidation in TS.

**Compound no**.	**Reactant**	**TS**	**Conjugation in TS (kcal mol^**−1**^)**
**12(Fer-1)** (active)	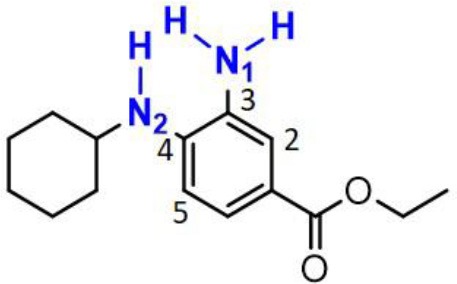	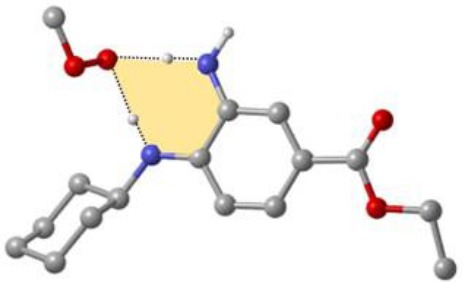	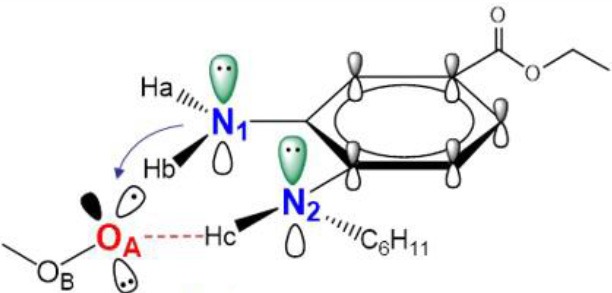
			φ = 9.01°, ψ = 3.81°
			${\text{n}}_{\text{N}1}\to \pi^{*}$ = 18.92
			${\text{n}}_{\text{N}2}\to \pi^{*}$ = 25.70
			*ΔG* = 10.45
**13** (inactive)	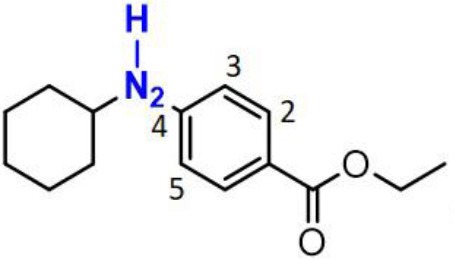	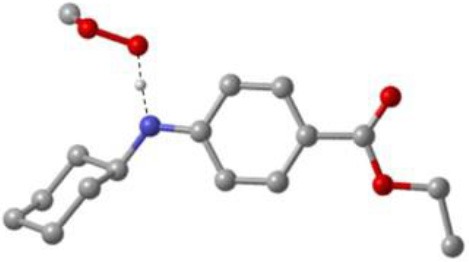	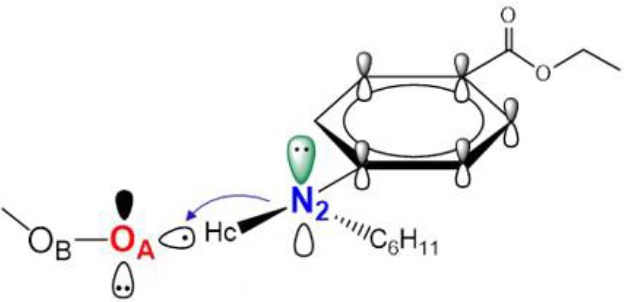
			ψ = 10.70°
			${\text{n}}_{\text{N}2}\to \pi^{*}$ = 3.6
			*ΔG* = 18.80

*φ = [(C_2_-C_3_-N_1_-H_a_) + (C_4_-C_3_-N_1_-H_b_)]/2; ψ = [(C_3_-C_4_-N_2_-H_c_) + (C_5_-C_4_-N_2_-C)]/2, Hydrogen atoms from C-H bond are omitted for clarity in TS structure*.

The type of radical-trapping mechanism of *ortho*-diamines is unique, which has not been found in other aminic RTAs or phenolic RTAs. Its inhibitory potency mainly depends on the following two aspects: (1) Compared with mono-aminic RTAs, presence of the *ortho*-NH can constraint twist the lone pair of reactive nitrogen (N_2_, **13**, Table 5) out of conjugation with the aromatic π-system. Hence, the extensive conjugation facilitates the electron transfer from nitrogen to the oxygen radical and accelerates the PCET reaction. (2) The formed planar seven-membered ring (φ = 9.01°, ψ = 3.81°) forces both -NH groups into better alignment with the aromatic π-system, with the energies of ${\text{n}}_{\text{N}}\to \pi^{*}$ interactions between two amines and benzene ring of 18.92 and 25.70 kcal mol^−1^ (Vatsadze et al., 2017), respectively. Furthermore, the *ortho*-NH acts as an acceptor that can interact with the orthogonal fully filled p-type orbital at the radical center via a classic H-bond type ${\text{n}}_{\text{O}}\to \sigma_{\text{H}4^{\text{′}}}^{*}$ interaction (Harris et al., 2016). Thus, one-electron oxidation in TS can be stabilized to a significant extent by the above two interactions. In summary, the *ortho*-diamine moiety provides a specific TS-stabilizing interaction to the N-H abstraction step.

Synergistic effect of *ortho*-diamines is a distinctive radical-trapping mechanism of aminic RTAs, but how to enhance this synergistic effect and to improve the potency are still lack of discussion. The NBO calculations were employed to measure the resonance stabilization of unpaired electron in transition states. As the data shown in Table 6, the tight interaction between *ortho*-diamines moiety and methylperoxyl radical (CH_3_OO•) promote the structure rearrangement and form a large conjugate system to effectively stabilize the unpaired electron. The conjugate system would be further strengthened by introducing a benzyl, pyridinyl, and other groups at position N_1_ (Skouta et al., 2014; Hofmans et al., 2016). The energies of ${\text{n}}_{\text{N}}\to \pi^{*}$ interactions between two amines and aromatic system are both increase to some extent which are attribute to maximize delocalization of the unpaired electron in the aminyl radical, minimizing the entropic cost. The calculated barrier of compound **14** and **15** with methylperoxyl radical are 9.62 and 9.57 kcal mol^−1^, much less than that (10.45 kcal mol^−1^) of compound **12**. Importantly, compound **14** and compound **15** present more potent than the parent compound (**12**) in erastin-induced ferroptosis (Skouta et al., 2014). Hence, the more extensive conjugation by the mono-substituted take place both at 3′NH and 4′-NH would be beneficial to stability to one-electron oxidation, eventually improves the radical scavenging activity.

**Table 6:** Synergetic effect between single substitution and double substitution on *ortho*-diamines.

**Compound no**.	**Reactant**	**TS *E(2)*/kcal·mol^**−1**^**	***Δ*G/kcal·mol^**−1**^**
**12(Fer-1)** (active)	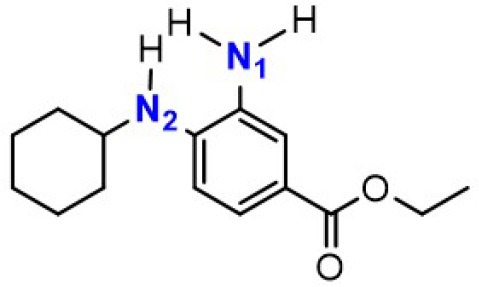	${\text{n}}_{\text{N}1}\to \pi^{*}$ = 18.92	0.45
		${\text{n}}_{\text{N}2}\to \pi^{*}$ = 25.70	
**14** (active)	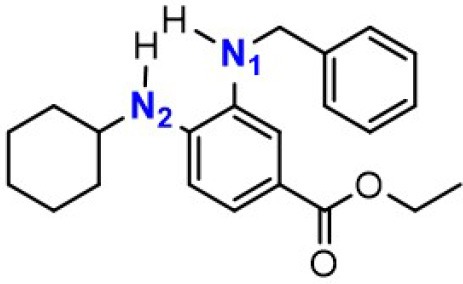	${\text{n}}_{\text{N}1}\to \pi^{*}$ = 19.67	9.62
		${\text{n}}_{\text{N}2}\to \pi^{*}$ = 26.04	
**15** (active)	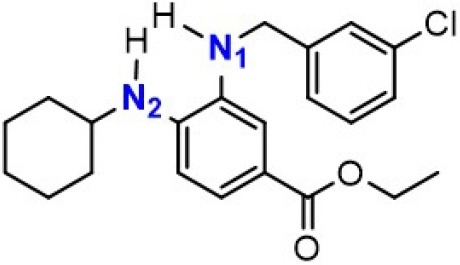	${\text{n}}_{\text{N}1}\to \pi^{*}$ = 19.66	9.57
		${\text{n}}_{\text{N}2}\to \pi^{*}$ = 26.21	

## Conclusions

In this work, we have discussed the mechanism of the “minor” type of radical trapping antioxidants (RTAs)—aminic RTAs by employing DFT calculations. The reaction of aminic RTAs with peroxyl radicals is a typical proton-coupled electron transfer (PCET) process, which is the most thermodynamically favorable pathway among the hydrogen atom transfers. And the following three structural features play a vital role in pharmacological activities of aminic RTAs:

1. *Stereoelectronic effect*: In a PCET reaction, the electron moves from the π-HOMO of the amine to the π-LUMO of the peroxyl radical. A good alignment of the p-type lone pair at reactive -NH with the aromatic π-system would lead to the increase of HOMO energy levels and finally remarkably increase the PCET rate. Thus, planar, intracyclic amine compounds are a good choice.
2. *Resonance effect:* The molecules containing highly conjugate structure make a significant contribution to resonance stabilization of the product radical and have a concomitant effect on the energy of the transition state. A π double bond with aromatic π system (or a strong p-π conjugation) is the minimum degree of resonance for the reactivity of bicyclic aminic RTAs.
3. *Ortho-diamine effect*: The synergistic effect of *ortho-diamine* moiety is a distinctive feature for aminic RTAs. A near-planar structure is formed by *ortho-diamine* and peroxyl radical in transition state (TS), which is favorable for unpaired electron delocalization through π-conjugation. The closer to the planar structure in TS, the more potency for the *ortho-diamine* RTAs.

**Figure 5:**
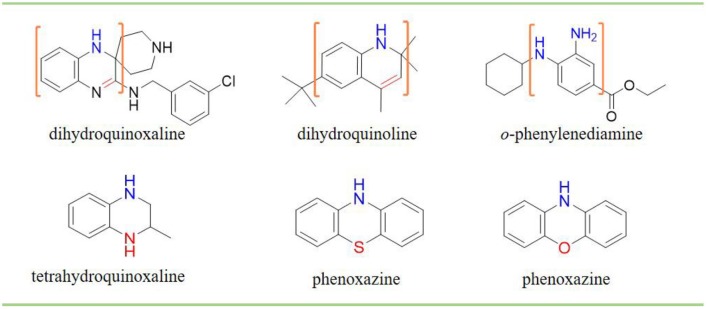
The main scaffold of aminic RTAs (inside the brackets are scaffold structures).

A trivalent nitrogen atom is potentially capable of offering more structural diversity of aminic RTAs (Figure 5) compared with classic phenolic RTAs. Such diversity should provide more ways to trap peroxyl radical. However, in fact, the types and amounts of aminic RTAs are far less than that of phenolic RTAs. We expect that aminic RTAs will receive more attention and more compounds for clinical use in future.

## Methods

All calculations were carried out with Gaussian 09 program (Frisch et al., 2009). The geometries of reactant, products and transition states were fully optimized at the MPWB1K functional (Lynch et al., 2000) with the 6 – 31 + G(d,p) basis set and characterized by the number of imaginary frequencies. We adopted the MPWB1K functional because it is more accurate than the traditional B3LYP method in describing the energy barrier of H-transfer reaction (Lingwood et al., 2006). Transition state structures were further confirmed by intrinsic reaction coordinate (IRC) calculation to connect the corresponding reactant and products. The geometry optimizations were undertaken without any symmetry constraints and carried outs at the same computation level. The discussed energies in this paper are referred to relative *Gibbs* free energies (Marković et al., 2011). In order to ensure that the lowest energy conformation of intermediates and transition states was presented and discussed in the text, extensive conformational searches were conducted. The natural bond orbital analysis (NBO) (Weinhold, 2012) was employed to investigate the bonding of each optimized molecules with the NBO6w and NBOpro program (Glendening et al., 2013). The 3D structures were prepared using *CYLview* (CYLview et al., 2009). Cartesian coordinates of all optimized structures are given in the ESI.

## Data Availability Statement

All datasets generated for this study are included in the article/Supplementary Material.

## Author Contributions

D-ZC, Z-HL, and X-HS contributed to the conception and design of the study. Y-ZL, X-LZ, and B-QH preformed the experiments and theoretical calculations. Y-ZL, X-LZ, and X-HS organized the database. Y-ZL, X-LZ, D-ZC, and X-HS wrote the first draft of the manuscript. All authors contributed to manuscript revision, read, and approved the submitted version.

### Conflict of Interest

The authors declare that the research was conducted in the absence of any commercial or financial relationships that could be construed as a potential conflict of interest.
