# Supplementary material for: Reactions of the Lipid Hydroperoxides With Aminic Antioxidants: The Influence of Stereoelectronic and Resonance Effects on Hydrogen Atom Transfer
Source: Front Chem. 2019 Dec 17;7:850. doi: 10.3389/fchem.2019.00850 (PMC6927943; doi:10.3389/fchem.2019.00850)
Supplement: Supplementary file 1 [file Table_1.DOCX]

**Re****actions of the Lipid Hydroperoxides with** **aminic antioxidants: The Influence of Stereoelectronic and Resonance Effects on Hydrogen Atom Transfer**

Yu-Zhen Li^†#^, Xiao-Lu Zhou^‡#^, Bao-Qi Huo^†^, De-Zhan Chen^†^, Zhao-Hua Liu^§*^, Xie-Huang Sheng^†*^

^†^College of Chemistry, Chemical Engineering and Materials Science, Collaborative Innovation Center of Functionalized Probes for Chemical Imaging in Universities of Shandong, Key Laboratory of Molecular and Nano Probes, Ministry of Education, Shandong Provincial Key Laboratory of Clean Production of Fine Chemicals, Shandong Normal University, Jinan 250014, P. R. China

^‡^Key Laboratory of Systems Bioengineering, Ministry of Education, Department of Pharmaceutical Engineering, School of Chemical Engineering and Technology, Tianjin University, Tianjin 300072, P. R. China

*^§^*Center for New Drug safety Evaluation, School of Pharmaceutical Sciences of Shandong University, Jinan, 250012, P. R. China

^#^ These authors contributed equally to this study.

**List of Contents**

| - Table S1 | 2-3 |
| --- | --- |
| - Table S2 | 4 |
| - The testing of anti-ferroptotic cell death on aminic RTAs | 5-7 |
| - Cartesian Coordinates(Å) of the Optimized Structures | 8-32 |

**Tab S1 The information of aminic RTAs**

| NO. | Structure | ΔG**/**kcal mol^-1^ | EC_50_ | Reference |
| --- | --- | --- | --- | --- |
| 1 |  | 11.83 | 70 | [1] |
| 2 |  | 14.43 | >12500 | This work |
| 3 |  | 7.08 | 10±2 | [1] |
| 4(Clozapine) |  | 13.99 | 12500 | This work |
| 5 |  | 18.32 | >1000 | [2] |
| 6(Lip-1) |  | 11.63 | 22 | [2] |
| 7 |  | 13.38 | >12500 | This work |
| 8 |  | 13.16 | > 12500 | This work |
| 9 |  | 12.49 | 1350 | [3] |
| 10 |  | 10.85 | 215 | [3] |
| 11 |  | 9.97 | 42 | [3] |
| 12(Fer-1) |  | 10.45 | 50 | [4] |
| 13 |  | 18.80 | >12500 | [5] |
| 14 |  | 9.62 | 6 | [4] |
| 15 |  | 9.57 | 41 | [4] |

Table S2 The conformation feature of Clozapine

| Reactant Conformation | TS Conformation |
| --- | --- |


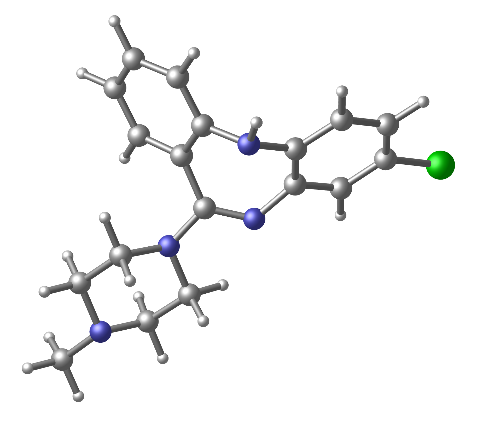

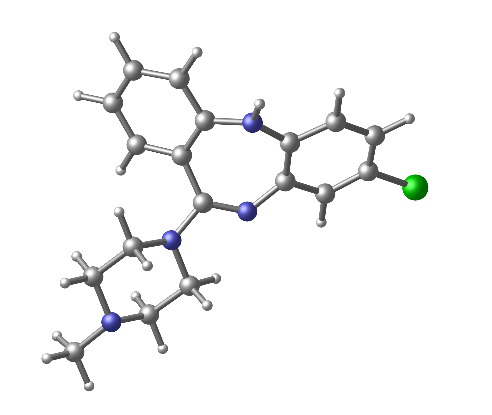


149˚

119˚

|  | *Δ*G = +6.93 Kcal mol^-1^ |
| --- | --- |

**The testing of anti-ferroptotic cell death on aminic RTAs**

**Methods**

Fer-1, Erastin, clozapine and olanzapine were obtained from Sigma-Aldrich (Sigma Chemicals St. Louis, MO, USA). Compound 2 was obtained from TCI Company. Compound 7 and 8 were purchased from Alfa Aesar (Ward Hill, MA, USA). Compound 18 and 19^[6]^ were kindly gifted from prof. Zhou Haifeng (China Three Gorges University). HT-1080^[7]^ cell line was obtained from the Cell Bank of the Chinese Academy of Sciences (Shanghai, China). The cells were cultured in MEM containing 10% FBS, 10 mM glutamine, 100 IU/mL penicillin, and 100 μg/mL streptomycin in a humidified incubator with 5% CO_2_ and 95% air. Cell death inhibition (EC_50_) values were determined by treating HT-1080 fibrosarcoma cells with a lethal concentration of erastin (10 μM) in the presence of each new synthesized compound in a 10-point, 2-fold dilution series for 48 h. Cell viability was assessed later using the MTT assay according to the manufacturer’s instructions. Each experiment was carried out in six analytical replicates per concentration and repeated independently at least three times.

**Results**

**Table S2**  Antiferroptotic Activity of aminic RTAs in Response to Erastin-Induced Ferroptosis in HT-1080 Human Fibrosarcoma Cells

| Compound NO. | Structure | EC_50_ (nM) |
| --- | --- | --- |
| 15 (Fer-1) |  | 50 ± 2 |
| 2 |  | >12500 |
| 4 (clozapine) |  | >12500 |
| 7 |  | >12500 |
| 8 |  | >12500 |
| 18 (olanzapine) |  | >12500 |
| 19 |  | 52 ± 3 |
| 20 |  | 73 ± 3 |

**References:**

1.Shah, R., K. Margison, and D.A. Pratt, *The Potency of Diarylamine Radical-Trapping Antioxidants as Inhibitors of Ferroptosis Underscores the Role of Autoxidation in the Mechanism of Cell Death.* ACS Chemical Biology, 2017. **12**(10): p. 2538-2545.

2.Friedmann Angeli, J.P., et al., *Inactivation of the ferroptosis regulator Gpx4 triggers acute renal failure in mice.* Nature cell biology, 2014. **16**(12): p. 1180-1191.

3.Li, W.-J., et al., *Mechanism-Guided Development of Tetrahydroquinoxaline Derivatives as Novel, Potent Ferroptosis Inhibitors* submited, 2019.

4.Skouta, R., et al., *Ferrostatins Inhibit Oxidative Lipid Damage and Cell Death in Diverse Disease Models.* Journal of the American Chemical Society, 2014. **136**(12): p. 4551-4556.

5.Sheng, X.-H., et al., *O-Phenylenediamine: a privileged pharmacophore of ferrostatins for radical-trapping reactivity in blocking ferroptosis.* Organic & Biomolecular Chemistry, 2018. **16**(21): p. 3952-3960.

6.Liu, S., et al., *B2(OH)4-mediated one-pot synthesis of tetrahydroquinoxalines from 2-amino(nitro)anilines and 1,2-dicarbonyl compounds in water.* Organic Chemistry Frontiers, 2017. **4**(11): p. 2175-2178.

7.Dixon, S.J., et al., *Ferroptosis: an iron-dependent form of nonapoptotic cell death.* Cell, 2012. **149**(5): p. 1060-72.

**Cartesian Coordinates(Å) of the Optimized Structures**

6-(tert-butyl)-2,2,4-trimethyl-1,2-dihydroquinoline **(1)**

Reactant Free Energy = -677.904790

0 1

C 1.43598000 -1.65426500 -0.21280600

C 0.07860400 -1.94178600 -0.32765800

C -0.87499900 -0.92352600 -0.28725600

C -0.43824500 0.41152300 -0.12901300

C 0.92982100 0.66375100 -0.01651700

C 1.89907900 -0.34589200 -0.04976700

H 2.13441400 -2.48181600 -0.24954900

H -0.24330700 -2.97175700 -0.45633300

H 1.24715400 1.69465300 0.10099300

C -2.75401300 1.12731300 -0.05949600

N -2.22055700 -1.17716000 -0.47538100

C -3.23378600 -0.29471500 0.10188300

C -3.45290200 -0.59646800 1.59755300

C -4.53841800 -0.50915600 -0.66848100

H -4.17616800 0.09878000 2.03452400

H -2.51161400 -0.49907000 2.14425000

H -5.33081300 0.13001500 -0.26927900

H -4.39924900 -0.27970800 -1.72701500

C -1.45638400 1.46860100 -0.13302100

H -3.83403300 -1.61480900 1.73569800

H -4.87468300 -1.54779000 -0.57953700

C 3.38305100 0.00879800 0.08624100

C 3.79559800 0.95680600 -1.05395900

C 3.62612400 0.70556700 1.43689700

C 4.28171600 -1.23077400 0.02122400

H 3.63807700 0.48552500 -2.02839400

H 3.22036500 1.88620100 -1.03645800

H 4.85533100 1.21986000 -0.96827900

H 3.34778000 0.05125000 2.26811100

H 4.68346400 0.96879200 1.54763900

H 3.04279300 1.62568100 1.52727200

H 5.32886300 -0.93038500 0.12185900

H 4.06209300 -1.93552100 0.82884900

H 4.17983600 -1.75713400 -0.93255900

C -1.02975500 2.90533000 -0.22230700

H -0.42936600 3.19774800 0.64595100

H -0.41896800 3.08481900 -1.11299600

H -1.89781400 3.56593700 -0.26445900

H -3.52068700 1.89828200 -0.05573700

H -2.46839000 -2.15607300 -0.44799100

Transition State Free Energy = -868.043601

0 2

C -1.38525400 1.52654000 -0.23345000

C -0.02532700 1.29439500 -0.29556000

C 0.48721200 -0.02091200 -0.16935700

C -0.43284000 -1.10053000 0.01860700

C -1.79476100 -0.81726900 0.06435000

C -2.30999000 0.48135100 -0.05551400

H -1.73235400 2.54783000 -0.33049900

H 0.67567800 2.11101300 -0.43165200

H -2.48298000 -1.64366900 0.20114400

C 1.44169700 -2.62055700 0.09595100

N 1.81155200 -0.22088800 -0.24793000

H 2.52434900 0.66407400 -0.26008000

C 2.44258500 -1.52133400 -0.08517100

C 3.36452100 -1.46150200 1.15313800

C 3.29754300 -1.78935400 -1.34088200

H 3.89394400 -2.41089100 1.27321900

H 2.77783000 -1.27062300 2.05428600

H 3.82971100 -2.73964600 -1.24143900

H 2.66551100 -1.83304200 -2.23044500

C 3.62936600 3.77331600 0.53510400

H 3.09627400 4.72539800 0.47192700

H 4.61620500 3.86359800 0.06884500

H 3.74608500 3.47394300 1.58240300

O 2.83937900 2.83950600 -0.16211700

O 3.49435200 1.60774800 -0.11670500

C 0.10797000 -2.44946000 0.14829600

H 4.09447000 -0.65676900 1.03646000

H 4.02919300 -0.98777800 -1.46997500

C -3.82113700 0.70933700 0.00467000

C -4.50105000 -0.05858500 -1.14384900

C -4.36521300 0.19663000 1.35083200

C -4.19094300 2.19076200 -0.12625200

H -4.13605400 0.28745200 -2.11503600

H -4.31592700 -1.13398000 -1.08022200

H -5.58450900 0.09542200 -1.11162600

H -3.90184900 0.72697900 2.18759200

H -5.44727800 0.35357900 1.40466700

H -4.17808100 -0.87168900 1.48661900

H -5.27777000 2.30145000 -0.07393800

H -3.75990700 2.79132100 0.68012600

H -3.86416700 2.61033900 -1.08218700

C -0.82330700 -3.61109700 0.34070200

H -1.41969200 -3.49827000 1.25206000

H -1.52291800 -3.70501200 -0.49632500

H -0.26460300 -4.54531600 0.41826600

H 1.86170600 -3.61792500 0.20220900

2-methylnaphthalen-1-amine **(2)**

Reactant Free Energy = -480.327564

0 1

C 1.49991500 -1.64403500 -0.01180100

C 0.16641300 -1.96768500 0.01125300

C -0.80694300 -0.94043200 0.01461400

C -0.38833800 0.42460200 -0.00468900

C 1.01249400 0.72367300 0.00767100

C 1.94877900 -0.30513000 -0.01180500

H -2.50305600 -2.27296600 0.04885000

H 2.24473300 -2.43466800 -0.02138300

H -0.15344500 -3.00441500 0.02232800

C -2.19468500 -1.23188100 0.02824600

C -1.38396500 1.43409400 -0.04187000

C -2.72280200 1.11829600 -0.03184100

C -3.13479000 -0.23069600 0.01123000

H -1.10010700 2.47893600 -0.10854600

H -3.46456700 1.90919100 -0.06694300

H -4.19220600 -0.47319200 0.02106100

N 1.43120400 2.05003300 -0.02009700

H 0.81502100 2.71525600 0.41887600

C 3.42010700 0.00124300 -0.02662300

H 3.70374200 0.60071400 -0.89969500

H 3.73622500 0.55430100 0.86785900

H 4.00774100 -0.91808000 -0.05606200

H 2.39038000 2.20239800 0.24802200

Transition State Free Energy = -670.462229

0 2

N -1.21893500 -0.85142600 -1.28366700

H -1.97841700 0.01844500 -1.04829300

C -0.07790400 -0.94804900 -0.57638000

C 0.37828900 -2.19419600 -0.06567600

C 0.72906700 0.24181000 -0.38134800

C 1.60031400 -2.24863700 0.61546900

C 1.95582400 0.14142800 0.33358600

C 2.37213900 -1.12197000 0.82428200

C -3.52562600 0.87203400 1.55303400

H -3.19546600 1.30720100 2.49885600

H -3.58970300 -0.21812800 1.64132600

H -4.50328900 1.27573800 1.27260300

O -2.54715100 1.23829000 0.60405900

O -2.93571000 0.74441600 -0.63233800

C 2.74092200 1.30256600 0.52724700

C 2.33517900 2.51830400 0.02423600

H 2.94452000 3.40261400 0.17764400

C 1.12903800 2.61174300 -0.69422100

H 0.81177700 3.56867000 -1.09420400

H 3.67284500 1.21669600 1.07768900

C 0.34200500 1.49524000 -0.89457700

H -0.58800600 1.58591500 -1.44166200

H -1.71319800 -1.73207500 -1.36433400

C -0.43833000 -3.43814900 -0.25762100

H -0.55351100 -3.69380600 -1.31747700

H 0.03589000 -4.29026200 0.23173900

H -1.44400900 -3.33376600 0.16615000

H 3.30935800 -1.19531300 1.36659100

H 1.94112300 -3.20633600 0.99708300

10*H*-phenoxazine **(3)**

Reactant Free Energy = -592.418947

0 1

C 3.62790500 0.64483500 0.00057900

C 2.43973900 1.37612300 0.00023800

C 1.20716500 0.72660100 -0.00027700

C 1.18415200 -0.67583700 -0.00027900

C 2.36101200 -1.40327300 0.00016200

C 3.59256900 -0.74409800 0.00053700

C -1.18415200 -0.67583600 -0.00030400

C -1.20716500 0.72660100 -0.00031600

C -2.43973900 1.37612300 0.00016000

H -2.46540500 2.46185000 0.00016200

C -3.62790500 0.64483400 0.00056500

C -3.59256900 -0.74409800 0.00059600

C -2.36101300 -1.40327300 0.00019000

H 4.57640300 1.17016500 0.00089500

H 2.46540400 2.46185000 0.00030200

H 2.29728400 -2.48546300 0.00011600

H 4.51091700 -1.31971200 0.00080600

H -4.57640200 1.17016800 0.00087900

H -4.51091800 -1.31971000 0.00091600

H -2.29728500 -2.48546200 0.00018600

N 0.00000000 1.40918900 -0.00081600

H 0.00000100 2.41393500 -0.00129800

O 0.00000000 -1.37801900 -0.00104600

Transition State Free Energy = -782.565326

0 2

C 3.19019000 -2.02176900 -0.14031200

C 1.82644400 -1.87180500 -0.34093100

C 1.22810800 -0.60886500 -0.21573100

C 2.04158400 0.49175900 0.11122600

C 3.40702000 0.34405100 0.30414900

C 3.98369900 -0.91630000 0.17954900

C 0.16451900 1.90684900 0.08048000

C -0.66477900 0.81641300 -0.24800600

C -2.04043000 1.04471000 -0.41033700

H -2.67568900 0.20227700 -0.66088800

C -2.56113500 2.31891400 -0.23885000

C -1.72313500 3.38893800 0.08551400

C -0.35565400 3.18142300 0.24373900

H 3.63984100 -3.00348500 -0.23684800

H 1.19864800 -2.71988500 -0.59145200

H 3.99645400 1.21864600 0.55381500

H 5.05044700 -1.03479800 0.33136000

H -3.62552600 2.48216100 -0.36480800

H -2.13164200 4.38497900 0.21275300

H 0.31863800 3.99153800 0.49620300

N -0.12056600 -0.43329800 -0.42210600

H -0.77088100 -1.32781500 -0.38398400

O 1.51262500 1.74580200 0.24477100

C -3.57121500 -2.85764600 0.75726800

H -4.61368600 -2.58456100 0.58067800

H -3.25893400 -2.54677800 1.75887000

H -3.43808600 -3.93771600 0.64697700

O -2.82071600 -2.17257000 -0.22652200

O -1.49152500 -2.49717200 -0.06755900

8-chloro-11-(4-methylpiperazin-1-yl)-5*H*-dibenzo[b,e][1,4]diazepine **(4)**

Reactant Free Energy = -1376.108003

0 1

C -1.69249000 -0.51550100 -0.24428900

C 0.61965700 -0.02677000 -0.07736200

C -2.20919200 0.65338000 -0.84068800

C 0.48505600 1.42072900 0.24314500

C -0.44644100 2.21946200 -0.43805500

C 1.29194700 2.00741600 1.22429900

H 1.98316100 1.37350500 1.76961500

C 1.23063300 3.37061200 1.48799500

C -2.59770600 -1.46451300 0.25111000

C -3.58788700 0.83452600 -0.93189400

H -2.20242700 -2.37416600 0.68580200

C -4.47994500 -0.11619700 -0.44575500

C -3.96573000 -1.25692700 0.15968100

C 2.08162300 -1.96437600 -0.19617500

C 2.97865300 0.20635200 -0.75362600

C 3.45206000 -2.36694600 0.32286500

H 1.96745300 -2.27612000 -1.24424000

H 1.29592300 -2.45534400 0.37695100

C 4.33318300 -0.22284400 -0.21829600

H 2.93743300 -0.00635100 -1.83283500

H 2.84478400 1.27839700 -0.61525500

H 3.58814000 -3.44116800 0.16340300

H 3.49417500 -2.18435200 1.41495000

H 5.11800000 0.29279800 -0.78083300

H 4.42237200 0.08649300 0.84249300

C 5.82429900 -2.08491400 0.06166700

H 5.93951500 -3.15681800 -0.11933900

H 6.59320400 -1.56064700 -0.51212100

H 6.00742600 -1.89612500 1.13603500

N -0.34935700 -0.85891100 -0.24866300

N -1.31709400 1.62132900 -1.36965700

N 1.91062300 -0.52369400 -0.07346100

N 4.51165700 -1.65472200 -0.37009200

H -1.81012500 2.31290000 -1.91808100

H -5.54952800 0.03152000 -0.52828000

H -3.97532100 1.73900400 -1.39272300

Cl -5.05820300 -2.46139100 0.79305200

C -0.49076600 3.59407800 -0.18715000

H -1.20316000 4.21053500 -0.72752900

C 0.35246400 4.16855200 0.75681900

H 0.30478000 5.23725400 0.93766800

H 1.86632700 3.80760700 2.24980300

Transition State Free Energy = -1566.243372

0 2

C -1.03416900 1.24594700 -0.25826000

C 1.13046800 0.22511900 -0.24091400

C -1.91655900 0.13068000 -0.12646600

C 0.81479500 -1.04945200 -0.92660700

C -0.41451300 -1.71598400 -0.72415100

C 1.77613700 -1.64096700 -1.75657100

H 2.69195100 -1.09355000 -1.95181200

C 1.58827300 -2.90306100 -2.30177100

C -1.59779700 2.52455700 -0.41190000

C -3.30682300 0.36866800 -0.03560000

H -0.92457400 3.36769500 -0.50260900

C -3.83647800 1.63555700 -0.16909900

C -2.96722300 2.71004300 -0.38918400

C 2.85342200 1.71242900 0.62348000

C 3.17857700 -0.67225100 0.81288300

C 4.35635500 1.87442700 0.46160400

H 2.57124200 1.83373300 1.67854000

H 2.32314500 2.47327800 0.05228800

C 4.67422400 -0.47807900 0.63960400

H 2.93832300 -0.64815100 1.88616600

H 2.87555600 -1.64106900 0.42058900

H 4.65330600 2.83801200 0.88693200

H 4.60384500 1.89675000 -0.61818300

H 5.19990600 -1.25810300 1.19913300

H 4.93864500 -0.59744500 -0.43027600

C 6.51389200 1.00635500 1.06950700

H 6.78398900 1.96980900 1.50938200

H 7.02062300 0.22175700 1.63747400

H 6.89553400 0.98258100 0.03193900

N 0.32286100 1.21508900 -0.05261400

N -1.52113100 -1.18123200 -0.10167300

N 2.44503000 0.40021200 0.13854000

N 5.08042500 0.81873800 1.14698600

H -2.20769200 -1.95872700 0.37873900

H -4.90628300 1.79696700 -0.12149400

H -3.95976500 -0.48295700 0.12017700

Cl -3.62072400 4.30848200 -0.59150300

C -0.56398700 -3.02876700 -1.22244800

H -1.49403200 -3.54725500 -1.01610200

C 0.42473300 -3.61354800 -1.99298600

H 0.27550500 -4.61453400 -2.38338900

H 2.34491500 -3.33615200 -2.94620700

C -4.46279000 -2.94002200 2.61924200

H -5.48370100 -2.58099200 2.76857700

H -3.79960200 -2.52960700 3.38768300

H -4.43733000 -4.03363400 2.65790000

O -4.07702300 -2.48613800 1.34066800

O -2.78606800 -2.94163200 1.09298800

Conformation (TS) Free Energy = -1376.096953

0 1

C -1.75585000 -0.37546600 -0.05346900

C 0.60258800 0.02153700 0.03711800

C -2.26313400 0.83091400 -0.62540500

C 0.60402200 1.47982700 0.29739300

C -0.35641100 2.33795200 -0.28367700

C 1.61209100 2.03541900 1.09615700

H 2.30875500 1.36266600 1.58458500

C 1.74500100 3.40819600 1.24858700

C -2.67567900 -1.36648400 0.33114000

C -3.64796500 0.92978100 -0.89035700

H -2.28547900 -2.28271400 0.75611700

C -4.53014000 -0.06494400 -0.52205000

C -4.03196900 -1.20394400 0.12065300

C 1.89787000 -2.03596500 -0.09237700

C 2.91232800 0.02191800 -0.84356900

C 3.26818100 -2.50972800 0.36484400

H 1.69369000 -2.39505700 -1.11063800

H 1.11947900 -2.43289600 0.55792900

C 4.26535400 -0.47622800 -0.36832100

H 2.77781500 -0.26357300 -1.89743800

H 2.86548800 1.10725800 -0.77901200

H 3.31386400 -3.59868300 0.26650700

H 3.39560700 -2.26682000 1.43836500

H 5.04378600 -0.05699000 -1.01340600

H 4.45065200 -0.11004900 0.66143100

C 5.63164700 -2.42932300 -0.06599400

H 5.65315100 -3.51586400 -0.18283200

H 6.39413400 -2.00370600 -0.72358100

H 5.90417900 -2.18999900 0.97861800

N -0.43432800 -0.74601700 -0.02112400

N -1.50617600 1.93314800 -0.92571600

N 1.84074900 -0.58039400 -0.04861900

N 4.32694100 -1.92382900 -0.43781000

H -1.84668800 2.58925600 -1.61542300

H -5.59161700 0.03784800 -0.71047200

H -4.01024600 1.82920700 -1.37579500

Cl -5.13414200 -2.45246600 0.62006500

C -0.16788800 3.73375000 -0.18243800

H -0.88539600 4.38006000 -0.67625700

C 0.86994500 4.25845800 0.56646100

H 0.97910700 5.33464600 0.64745600

H 2.52990100 3.81293800 1.87746600

*N*-(2-chlorobenzyl)-9-methyl-1,4-diazaspiro[5.5]undec-4-en-5-amine **(5)**

Reactant Free Energy = -1286.256014

0 1

C -1.69206300 2.80675600 0.86133100

C -0.59200500 3.29307100 -0.06048800

C -0.33502400 1.04370800 -0.72675100

N -2.46355900 1.81691600 0.13042500

H -3.30711800 1.57311200 0.63214100

N 0.17224500 2.21694900 -0.65097000

N 0.39160900 0.04924100 -1.34366000

C -1.71586900 0.60535800 -0.21312900

C -2.50312500 -0.12423900 -1.32117200

C -1.54052300 -0.35256700 0.99676100

C -3.83298000 -0.68506500 -0.81920900

H -1.90896300 -0.95484500 -1.71679900

H -2.66309700 0.57661000 -2.14494600

C -2.87013600 -0.93111900 1.48219900

H -0.87942200 -1.18278300 0.72009700

H -1.03376700 0.17690200 1.81010000

H -4.33586800 -1.20874100 -1.64022200

H -4.50737400 0.13312600 -0.53195400

H -2.68167100 -1.62856400 2.30634500

H -3.49937700 -0.13605100 1.90822500

H 0.11671900 -0.90285300 -1.17051200

C 1.78181300 0.24875600 -1.69745000

H 2.03258900 -0.47216600 -2.47975700

H 1.87110700 1.25220600 -2.11434100

C 2.74545600 0.10698200 -0.53961100

C 3.08676100 -1.13574900 0.00187200

C 3.33217400 1.23646300 0.03916000

C 3.97642000 -1.26668700 1.06292800

C 4.22332500 1.13038300 1.10257100

C 4.54708000 -0.12337700 1.61440400

H 4.66211000 2.02477200 1.53158100

H 5.24198600 -0.21844200 2.44187700

H 3.05838200 2.20921900 -0.35475200

H 4.21666400 -2.25149100 1.44597200

Cl 2.38427500 -2.59964600 -0.66285000

H -1.03087300 3.89958600 -0.86388100

H 0.10053900 3.94390100 0.48552100

H -1.24575800 2.41692000 1.79329700

H -2.35489300 3.63253700 1.13646800

C -3.64695600 -1.63489500 0.36683100

H -3.03368400 -2.48111900 0.02039100

C -4.97766800 -2.18635800 0.86775500

H -5.51519200 -2.71240600 0.07294900

H -4.83310300 -2.88759700 1.69538900

H -5.62476000 -1.37811900 1.22668100

Transition State Free Energy = -1476.379177

0 2

C 2.86771300 -1.52732700 1.49980000

C 1.65325200 -1.41310500 2.42430900

C 0.49210600 -0.29399800 0.75143200

N 2.86615200 -0.63344600 0.36988000

H 3.01019400 -1.25778500 -0.65470100

N 0.42631100 -1.13419500 1.71654600

N -0.65341800 0.09690500 0.10172000

C 1.80680200 0.36431000 0.30011800

C 1.77357800 0.92833100 -1.13281500

C 2.12979900 1.54089100 1.26501000

C 3.08285300 1.62526800 -1.50891500

H 0.95936700 1.65789100 -1.20375000

H 1.56467500 0.12178500 -1.84337600

C 3.42607300 2.25979200 0.88999200

H 1.27722700 2.22812600 1.22933400

H 2.19020600 1.18174500 2.29463300

H 3.00643500 1.99835200 -2.53618000

H 3.89261900 0.88927500 -1.50375900

H 3.58674100 3.09081800 1.58642500

H 4.26381700 1.56689400 1.02546300

H -0.53117000 0.42538700 -0.84295600

C -1.90388100 -0.58398800 0.36455700

H -1.97743500 -1.51044300 -0.21714600

H -1.88375900 -0.89372700 1.41360200

C -3.10353000 0.28956400 0.09351100

C -4.37226800 -0.26025300 -0.12570100

C -3.00596200 1.68452800 0.08173500

C -5.49066300 0.53350900 -0.36069000

C -4.11358700 2.49438200 -0.14888800

C -5.35995500 1.91817800 -0.37490100

H -4.00162600 3.57323700 -0.14944700

H -6.23071700 2.53790500 -0.55896500

H -2.03466100 2.12972500 0.26783700

C 1.83896400 -4.03582700 -1.33515700

H 2.05186800 -5.04255400 -0.96862300

H 1.60649800 -4.06811800 -2.40345100

H 0.99780200 -3.60327700 -0.78376000

O 3.02330600 -3.29389000 -1.11219000

O 2.82214500 -2.00611800 -1.60990400

H 1.53380100 -2.35371500 2.97026900

H 1.82701700 -0.64721500 3.19241500

H 3.80069900 -1.38340600 2.06579400

H 2.94194100 -2.54242700 1.08127300

H -6.45201900 0.06249800 -0.52820400

Cl -4.59630200 -1.99330100 -0.09373200

C 3.43535000 2.77488800 -0.55539200

H 4.45942000 3.09384700 -0.78627700

C 2.53259900 3.99586600 -0.74538300

H 2.83866400 4.81291700 -0.08492800

H 2.58524700 4.36312600 -1.77484600

H 1.48190200 3.78041500 -0.52923200

*N*-(3-chlorobenzyl)-1*'H*-1λ²-spiro[piperidine-4,2'-quinoxalin]-3'-amine **(6)**

Reactant Free Energy = -1415.405737

0 1

C 4.79225600 -2.48512700 0.37786800

C 4.56702300 -1.15116400 0.04618900

C 3.28127400 -0.71882700 -0.28055700

C 2.20953500 -1.63454500 -0.29300900

C 2.45401000 -2.96693400 0.04632200

C 3.73425200 -3.39387000 0.38718400

H 5.79518100 -2.81058100 0.63302800

H 5.39068300 -0.44195200 0.03899100

H 1.62018700 -3.66064200 0.01965800

H 3.90818200 -4.43207200 0.64802400

C 0.69252000 0.02420600 -0.81371100

N 2.98860400 0.58413200 -0.67025200

H 3.74316500 1.23853200 -0.53017700

N 0.94013900 -1.23817900 -0.70348000

N -0.49454600 0.44028900 -1.33158100

C 1.66819200 1.10279500 -0.32388600

C 1.50576100 2.47293100 -0.99972500

C 1.46921400 1.27695500 1.20383800

C 2.41072800 3.53419000 -0.35716500

H 0.47563400 2.82949300 -0.88042100

H 1.70818000 2.38081000 -2.07165000

C 2.37247100 2.37774700 1.76452400

H 0.41815600 1.52581400 1.39887700

H 1.67283100 0.32404800 1.70350900

H 2.21830300 4.50722700 -0.81790100

H 3.46727600 3.31589300 -0.56235400

H 2.16738300 2.52782800 2.82800000

H 3.42177400 2.06194900 1.70070800

N 2.25038100 3.65912900 1.08217700

H 1.35426200 4.08379400 1.29690200

H -0.73458300 1.41187100 -1.24132400

C -1.55736800 -0.47293800 -1.71022800

H -2.02509100 -0.09730000 -2.62508900

H -1.07975200 -1.42741500 -1.93844300

C -2.60275500 -0.65008400 -0.63193000

C -3.87251600 -0.09186200 -0.78427000

C -2.30252000 -1.35942200 0.53669600

C -4.82036500 -0.24114400 0.22479800

C -3.26013700 -1.49841500 1.53520100

C -4.52949200 -0.93977800 1.38989600

H -3.02455900 -2.05285500 2.43756600

H -5.28107300 -1.04726000 2.16277300

H -1.31660400 -1.79822600 0.64827600

H -4.13078800 0.45418300 -1.68574700

Cl -6.40195900 0.46358700 0.01709100

Transition State Free Energy = -1605.544867

0 2

C -4.14456400 -2.77627700 -0.94707100

C -3.97982600 -1.42519300 -0.71505500

C -2.69262500 -0.90474700 -0.44292100

C -1.58228600 -1.79448700 -0.32304000

C -1.77572700 -3.15525300 -0.58663600

C -3.03694600 -3.64066200 -0.90346800

H -5.12958300 -3.16866500 -1.17462000

H -4.81637700 -0.73760500 -0.75738100

H -0.92349700 -3.82021400 -0.49742000

H -3.17153100 -4.69917500 -1.09932700

C -0.18751000 -0.07308200 0.28145600

N -2.50838200 0.42925000 -0.27693100

H -3.24850700 0.96189200 0.43396100

N -0.36731500 -1.35413400 0.15474300

N 0.91584800 0.38254100 0.91492300

C -1.14680800 0.94865000 -0.32498500

C -1.12587800 2.33597700 0.34032400

C -0.74423500 1.11699600 -1.82082600

C -1.97296300 3.35138800 -0.44056100

H -0.09868800 2.72156400 0.35221100

H -1.47172500 2.25823700 1.37598300

C -1.59723400 2.18269000 -2.51357100

H 0.31553700 1.39934100 -1.86103800

H -0.84487000 0.15214700 -2.32871700

H -1.88719800 4.33340500 0.03290000

H -3.02967500 3.07336700 -0.38913300

H -1.24899700 2.32122500 -3.54135900

H -2.63348100 1.83318700 -2.57131200

N -1.59908600 3.47199700 -1.84086000

H -0.68809200 3.91174200 -1.92473500

H 1.08299000 1.37380500 0.92331100

C 1.94327200 -0.47936900 1.47108300

H 2.20932100 -0.10581200 2.46425500

H 1.49002200 -1.46548300 1.58874000

C 3.17809200 -0.56606400 0.60332200

C 4.39625500 -0.06314100 1.05962400

C 3.10970700 -1.15026900 -0.66629600

C 5.52609400 -0.14546300 0.24927300

C 4.24594600 -1.22295800 -1.46389400

C 5.46649700 -0.72074400 -1.01381400

H 4.18999600 -1.68064400 -2.44592000

H 6.35620900 -0.77660500 -1.62941300

H 2.16366700 -1.55056400 -1.01563900

C -4.60664200 -0.06987500 3.04763400

H -5.36932200 -0.83413300 3.21149400

H -4.72277000 0.74186900 3.77204200

H -3.60790100 -0.50889300 3.13937800

O -4.82206300 0.40965600 1.73548800

O -3.90184100 1.41238300 1.47699500

H 4.47375900 0.38861000 2.04305500

Cl 7.03992900 0.48851800 0.83483700

2,2,4,7-tetramethyl-1,2,3,4-tetrahydroquinoline **(7)**

Reactant Free Energy = -561.264505

0 1

C 2.83107300 -0.48792800 -0.02943500

C 1.64471800 -1.18286800 -0.23406900

C 0.39099800 -0.55254000 -0.17137900

C 0.31844800 0.82780300 0.10363200

C 1.51891400 1.50894100 0.31863800

C 2.75631700 0.87934000 0.25821400

H 1.68113200 -2.24746400 -0.45573700

H 1.48675300 2.56917300 0.54935200

C -2.13692400 0.65787300 -0.38836500

N -0.75519300 -1.29760900 -0.42110000

C -2.05424400 -0.81152500 0.03487400

H -0.62672100 -2.29452600 -0.32068000

H -2.07581200 0.69395300 -1.48292800

C -1.02544300 1.52825700 0.20307400

H -1.24784600 1.68128800 1.26953400

H -3.11394200 1.06428500 -0.10531100

C -2.20926400 -0.96837200 1.55606200

H -3.15207100 -0.53316900 1.90266000

H -2.20607400 -2.02774800 1.83204200

H -1.38959500 -0.48333900 2.09148600

C -3.13446700 -1.62037700 -0.68003400

H -3.04663400 -2.68621500 -0.44242900

H -4.13023900 -1.29376400 -0.36783300

H -3.04988000 -1.50394100 -1.76337900

C -1.04110700 2.90709900 -0.46187100

H -0.34615000 3.60408700 0.01136600

H -0.77195600 2.82990300 -1.51960600

H -2.04050200 3.34868000 -0.39531700

C 4.16023100 -1.18442900 -0.13846200

H 4.60982600 -1.01832200 -1.12345600

H 4.86673000 -0.81197200 0.60809600

H 4.06009300 -2.26376700 -0.00072200

H 3.66372900 1.44846700 0.43529400

Transition State Free Energy = -751.399841

0 2

C 0.54807400 2.76483600 -0.22413300

C 0.80673600 1.42824200 -0.47016000

C -0.17316300 0.42337000 -0.24255300

C -1.47061900 0.79487200 0.22230200

C -1.70896500 2.14556500 0.44852700

C -0.72940600 3.11636900 0.24194900

H 1.78209200 1.11131900 -0.82323300

H -2.68629400 2.46188300 0.79649500

C -2.16637800 -1.51738500 -0.35731800

N 0.17256400 -0.85799000 -0.51293700

H 1.30623600 -1.14693000 -0.55382400

C -0.71481700 -1.96643700 -0.16649000

C 4.11920000 -0.76751800 0.99102200

H 4.77983300 0.09758100 1.08873100

H 4.71144500 -1.67895500 0.86027100

H 3.49041300 -0.86378600 1.88307200

O 3.33164000 -0.52393100 -0.15201300

O 2.49517500 -1.62535800 -0.34106300

H -2.32859300 -1.31341900 -1.42345300

C -2.52394900 -0.26842900 0.44664200

H -2.50722100 -0.53546000 1.51356900

C -3.94510300 0.19162500 0.11819000

H -4.28403400 0.99750400 0.77299300

H -4.01402900 0.54287900 -0.91573400

H -4.64426400 -0.64059000 0.24152200

H -2.83288000 -2.34238100 -0.08435900

C -0.44248700 -2.39620200 1.28652100

H -1.11567700 -3.21111200 1.57010600

H 0.58929000 -2.74079400 1.38191800

H -0.58566300 -1.56809200 1.98471600

C -0.39289800 -3.12768800 -1.10938500

H 0.65448400 -3.42419300 -1.01413800

H -1.01916500 -3.99207400 -0.87141900

H -0.57436400 -2.84288000 -2.14899900

H -0.96342200 4.15914400 0.43522800

C 1.59624600 3.81818700 -0.45464600

H 2.52900600 3.37751200 -0.81085800

H 1.26326300 4.54964100 -1.19742200

H 1.81226200 4.36699600 0.46716500

6-bromo-2-methyl-1,2,3,4-tetrahydroquinoline **(8)**

Reactant Free Energy = -3014.711562

0 1

C 1.09428100 1.51310500 -0.11501200

C -0.25410100 1.81576700 -0.22593800

C -1.22823700 0.80511300 -0.24543300

C -0.81848000 -0.53933000 -0.14054700

C 0.54095100 -0.83125700 -0.04188600

C 1.48880500 0.18243000 -0.02673500

H -0.56217000 2.85408900 -0.31044900

H 0.85764300 -1.86682400 0.02727700

C -3.17055600 -1.17933500 -0.73815400

N -2.56306500 1.13218100 -0.41966600

H -2.79611200 2.09186600 -0.21698100

C -3.61077500 0.16079900 -0.14636400

H -3.94969700 -1.92896500 -0.57275300

H -3.05862700 -1.05480000 -1.82006300

C -1.84653800 -1.64354100 -0.13680800

H -1.46598600 -2.50641000 -0.69238600

H -2.00642100 -1.99428200 0.89094600

C -3.96367700 0.07318800 1.34075900

H -4.32142900 1.03871100 1.71029700

H -4.75477900 -0.66398500 1.51068900

H -3.09473600 -0.20899100 1.94091400

H -4.50007400 0.49607500 -0.69246900

H 1.83177600 2.30674200 -0.10286000

Br 3.32491500 -0.24657500 0.11777700

Transition State Free Energy = -3204.848237

0 2

C -1.25259700 1.08330600 -0.70493200

C 0.11434600 0.92933600 -0.81343800

C 0.75660200 -0.24266000 -0.33449400

C -0.03287000 -1.26724100 0.26403700

C -1.40653100 -1.09684900 0.35583900

C -2.01057300 0.06406800 -0.12417100

H 0.72404600 1.70646800 -1.25895700

H -2.01464400 -1.86978700 0.81369300

C 2.09323000 -2.20233600 1.15000500

N 2.09876800 -0.34742900 -0.48679000

H 2.74783800 0.63711600 -0.47001500

C 2.81368100 -1.52737900 -0.01754000

C 2.74235000 3.44024400 1.00425800

H 2.10764600 4.31889100 0.86887900

H 3.77399200 3.74747400 1.20033600

H 2.37380700 2.83220100 1.83687600

O 2.66675700 2.72273800 -0.20987400

O 3.48667100 1.60463200 -0.10314500

H 2.62052200 -3.11875700 1.43005700

H 2.12380500 -1.53134300 2.01537200

C 0.64162100 -2.50210100 0.79263300

H 0.09711600 -2.87886600 1.66349600

H 0.59729200 -3.29733200 0.03735900

C 3.06524700 -2.46722200 -1.20207800

H 3.61093700 -1.94301500 -1.98916200

H 3.65942000 -3.32793100 -0.88169600

H 2.12893000 -2.83189400 -1.63209100

H 3.78677200 -1.16480500 0.33071700

H -1.73554200 1.98081400 -1.07171500

Br -3.87622200 0.26533800 0.02279500

2-methyl-3,4-dihydro-2H-benzo[*b*][1,4]thiazine **(9)**

Reactant Free Energy = -802.321339

0 1

C -3.18349800 0.43846500 0.01961400

C -2.09544500 1.29885200 0.05261100

C -0.77799900 0.81641600 0.03560400

C -0.57637400 -0.57645900 -0.00659000

C -1.68053200 -1.43252900 -0.03508700

C -2.97976700 -0.93968400 -0.02890000

H -2.25536200 2.37311600 0.09517400

H -1.50898700 -2.50439600 -0.05923500

C 2.09856300 0.09726300 0.37842900

N 0.28038000 1.71395700 0.11831200

H 0.01957900 2.66674100 -0.08073400

C 1.60439800 1.34845800 -0.33409800

H 2.02786000 0.26048100 1.45772900

H -3.81986500 -1.62436900 -0.05442000

H -4.18868500 0.84595900 0.03159300

H 1.65398600 1.18243600 -1.42136800

H 2.27606600 2.17859700 -0.09105900

S 1.03291200 -1.32061600 -0.05356300

C 3.52787400 -0.25470800 -0.00606400

H 4.20690800 0.54708000 0.29932100

H 3.85468600 -1.17440500 0.48513700

H 3.62124700 -0.39552900 -1.08643200

Transition State Free Energy = -992.459095

0 2

C 0.99475800 2.89423500 -0.26132000

C 1.15566100 1.56875800 -0.60420400

C 0.08598700 0.64012400 -0.47858800

C -1.15081800 1.10679500 0.05934200

C -1.29362700 2.45772700 0.38196600

C -0.24058400 3.34959400 0.22003000

H 2.09883900 1.19857900 -0.98878700

H -2.23915400 2.80432500 0.78673200

C -1.66020300 -1.56653100 0.32093300

N 0.30093200 -0.62516400 -0.91265600

H 1.37499700 -1.07947800 -0.80999800

C -0.73929600 -1.62626400 -0.88866400

C 3.80136700 -1.02771000 1.23135300

H 4.54618200 -0.25587500 1.43767700

H 4.26402700 -2.01841200 1.27228700

H 2.98626600 -0.97020600 1.96015800

O 3.32578400 -0.76159600 -0.07138500

O 2.39557100 -1.73929900 -0.40191200

H -1.05966400 -1.62431300 1.23461700

H -0.37616600 4.39310800 0.48188200

H 1.82342700 3.58432000 -0.37498100

H -1.34281400 -1.53646400 -1.80349800

H -0.24423200 -2.60224200 -0.92334700

S -2.52219800 0.04165600 0.32812900

C -2.69940100 -2.67688600 0.30826900

H -2.20213700 -3.65003100 0.35042600

H -3.36943100 -2.60526400 1.16834800

H -3.30541400 -2.64029700 -0.60130900

2,2-dimethyl-3,4-dihydro-2H-benzo[*b*][1,4]oxazine **(10)**

Reactant Free Energy = -518.605532

0 1

C -3.14904600 0.27457000 0.14223000

C -2.11876200 1.20357900 0.06275200

C -0.79272400 0.79554500 -0.12073300

C -0.51955300 -0.58080300 -0.20161500

C -1.55640300 -1.50933800 -0.14917800

C -2.87043600 -1.08724400 0.01929500

H -2.33146400 2.26712500 0.12066100

H -1.30500500 -2.56174500 -0.22359800

C 1.81401500 -0.17031800 0.05687800

N 0.24494500 1.72236300 -0.24746400

H 0.23100200 2.47253900 0.42512800

C 1.54799700 1.19678200 -0.58836300

H -3.67014400 -1.81825000 0.06395800

H 1.61999300 1.07466800 -1.67632000

H 2.31542000 1.91332300 -0.28672800

O 0.75468900 -1.05669500 -0.34650500

H -4.16917200 0.61490000 0.28304100

C 1.85311000 -0.08369200 1.57927200

H 1.99546200 -1.07927400 2.00590300

H 2.68008400 0.55378300 1.90545700

H 0.92298400 0.32685500 1.97952000

C 3.08385400 -0.79143200 -0.49798700

H 3.95588000 -0.18846500 -0.23084500

H 3.22044900 -1.79577300 -0.09063300

H 3.03006700 -0.86856600 -1.58656600

Transition State Free Energy = -708.745893

0 2

C 0.77780500 2.98862600 -0.15596300

C 1.03568000 1.70665700 -0.59966700

C 0.03017800 0.71050200 -0.54600000

C -1.23066800 1.04897900 0.02034000

C -1.48565800 2.35072500 0.43768800

C -0.48939100 3.31746200 0.34824300

H 2.00114400 1.43018100 -1.00737400

H -2.46262400 2.58039700 0.84772600

C -1.80353100 -1.26115600 0.12543800

N 0.25452700 -0.52702800 -1.05254900

H 1.30298100 -1.00717700 -0.94568200

C -0.85708300 -1.44962900 -1.06148700

C 3.73844200 -0.94347400 1.10563700

H 4.46077700 -0.15223700 1.31810900

H 4.23171200 -1.91986900 1.12649200

H 2.92904700 -0.92318300 1.84275400

O 3.24282500 -0.67169600 -0.18862100

O 2.33873100 -1.66871500 -0.52706800

H -0.69538900 4.32844600 0.68238100

H -1.42482900 -1.30602500 -1.99144900

H -0.46199600 -2.46849300 -1.07397500

O -2.21380700 0.12819600 0.14851700

H 1.55303600 3.74460600 -0.21052700

C -1.13393300 -1.60350800 1.45120300

H -1.81211800 -1.38243800 2.27852800

H -0.87989500 -2.66610600 1.48104200

H -0.21204200 -1.03602300 1.59441000

C -3.08627800 -2.04817400 -0.07229200

H -2.87330900 -3.11956500 -0.10517000

H -3.77592900 -1.85867800 0.75328900

H -3.57762000 -1.75898700 -1.00417400

2-methyl-1,2,3,4-tetrahydroquinoxaline **(11)**

Reactant Free Energy = -498.736768

0 1

C 2.98083600 -0.61911200 0.31834000

C 1.77063200 -1.23128300 0.63433800

C 0.55064200 -0.62662700 0.32527300

C 0.55064600 0.62664100 -0.32526200

C 1.77063800 1.23130500 -0.63431200

C 2.98083700 0.61913200 -0.31831400

H 1.76383400 -2.19341100 1.13989900

H 1.76382100 2.19343500 -1.13987200

C -1.87248900 0.76884300 -0.00806200

N -0.66696800 -1.19677000 0.70181600

H -0.61897300 -2.18746000 0.88739100

C -1.87249600 -0.76884500 0.00801800

H -2.72347200 -1.09157400 0.61867500

H -2.72345700 1.09157000 -0.61872800

C -2.01431900 -1.36152700 -1.39393000

H -2.00497100 -2.45467800 -1.35063900

H -2.95796300 -1.05153500 -1.85396500

H -1.19523900 -1.03380500 -2.03789700

C -2.01445700 1.36147800 1.39391400

H -2.00479100 2.45463000 1.35069000

H -2.95830400 1.05171300 1.85368100

H -1.19559900 1.03349600 2.03802800

N -0.66696100 1.19679200 -0.70177200

H -0.61896500 2.18742400 -0.88762800

H 3.91437700 -1.10981400 0.57077000

H 3.91438400 1.10983400 -0.57072500

Transition State Free Energy = -688.878527

0 2

C -0.30897500 2.97431000 -0.43566100

C 0.31083500 1.77216000 -0.70963300

C -0.33109600 0.54345400 -0.42132900

C -1.62090600 0.56739500 0.19475800

C -2.23920300 1.79913100 0.43862100

C -1.59489000 2.98797800 0.12831200

H 1.29920800 1.73269300 -1.15315300

H -3.22661000 1.81415500 0.89134700

C -1.37872700 -1.80736400 0.66982700

N 0.26429700 -0.63054800 -0.74329800

H 1.39340100 -0.71679200 -0.71014200

C -0.45480500 -1.87968500 -0.55530100

C 3.91327300 0.44405200 0.93614400

H 4.36151100 1.44033400 0.92040400

H 4.69344800 -0.31859600 1.02360200

H 3.21805200 0.35661200 1.77798100

O 3.22991400 0.30892000 -0.29085000

O 2.66240300 -0.95787200 -0.33575400

H 0.30290900 -2.64674200 -0.36603300

H -2.03928300 -2.68045500 0.64761700

C -1.21198300 -2.24303900 -1.83438500

H -0.52454800 -2.26744200 -2.68200200

H -1.67720600 -3.22844400 -1.73541400

H -1.99450500 -1.50973300 -2.04263600

C -0.60129600 -1.79717200 1.98530100

H -1.28160700 -1.71025100 2.83737000

H -0.02964200 -2.72245000 2.10021500

H 0.10149300 -0.96105400 2.01849100

N -2.22302000 -0.63059700 0.50380500

H -3.02097500 -0.56814900 1.11791500

H 0.19272300 3.90688900 -0.66789000

H -2.08919900 3.93173400 0.33168000

ethyl 3-amino-4-(cyclohexylamino)benzoate **(12)**

Reactant Free Energy = -844.390488

0 1

C 1.38340400 -0.65610800 -0.04023500

C 2.20554800 0.46893900 0.02064500

C 1.61918400 1.73748600 0.13153400

C 0.24529700 1.89124200 0.16183300

C -0.59412400 0.75054800 0.05378600

C 0.00057200 -0.51614700 -0.02107400

H 1.82543200 -1.64224100 -0.11073400

H 2.27124800 2.60253600 0.20394200

H -0.61982300 -1.40161700 -0.08500300

N -0.38111700 3.15220700 0.22446800

H 0.28304400 3.91269400 0.26753300

H -1.03074000 3.22722500 1.00003200

N -1.95689400 0.96821000 0.04549700

H -2.19686900 1.87973300 -0.32462300

C 3.67853000 0.38921700 -0.00489200

O 4.42349700 1.34966100 0.04642800

O 4.13278000 -0.87666500 -0.09197200

C 5.55837800 -1.02801600 -0.12012900

H 5.95647000 -0.47065100 -0.97252600

H 5.97856600 -0.58526700 0.78719400

C 5.85933500 -2.50501600 -0.22009700

H 5.43384000 -2.93133900 -1.13168000

H 6.94115400 -2.66072100 -0.24177400

H 5.45247500 -3.04693600 0.63695400

C -2.92609600 -0.08058200 -0.22440300

C -3.26480600 -0.87327300 1.04287000

C -4.19546900 0.53342000 -0.81445400

H -2.51521400 -0.77687100 -0.97432400

C -4.31033800 -1.95189400 0.76359500

H -3.64663800 -0.16539700 1.78949300

H -2.35750100 -1.31294000 1.46820500

C -5.25777200 -0.53056500 -1.09104600

H -4.59134800 1.27197500 -0.10345600

H -3.95078600 1.07256300 -1.73721800

C -5.57795000 -1.34910900 0.15938000

H -4.54596400 -2.49122100 1.68680600

H -3.89475400 -2.69462500 0.06902900

H -6.16421500 -0.05528400 -1.47996900

H -4.89673600 -1.20261000 -1.88065800

H -6.30006500 -2.13677700 -0.08030500

H -6.05788600 -0.69996700 0.90379500

Transition State Free Energy = -1034.531497

0 2

C 2.04155100 -0.92615600 -0.10038900

C 2.67047900 0.33482800 -0.17457500

C 1.90076700 1.46543500 -0.38738900

C 0.50900300 1.39294600 -0.56191300

C -0.14126400 0.10420500 -0.45850400

C 0.67628400 -1.03202700 -0.23194200

H 2.64053400 -1.81129900 0.07275900

H 2.39772500 2.42874200 -0.44505200

H 0.21291000 -2.00644600 -0.14987100

N -0.20461000 2.49870600 -0.89273500

H 0.30338600 3.36953300 -0.83449100

H -1.22929800 2.57347600 -0.65367100

N -1.47852400 0.02785600 -0.58724700

H -2.03126400 0.90868200 -0.58276100

C 4.13255700 0.50930600 -0.02942600

O 4.70506700 1.57955900 -0.08584800

O 4.77373000 -0.65472400 0.17452700

C 6.19970900 -0.56609900 0.32300500

H 6.61738100 -0.10467300 -0.57585400

H 6.42408200 0.09206100 1.16664400

C 6.72514300 -1.96524500 0.53890000

H 6.48861300 -2.60898000 -0.31151400

H 7.81169300 -1.93613000 0.65419400

H 6.29755300 -2.41092800 1.44007200

C -2.26524300 -1.18533400 -0.44330800

C -2.53190600 -1.52459100 1.03080800

C -3.58149400 -1.02067700 -1.20598700

H -1.70965900 -2.01227300 -0.90426300

C -3.40412500 -2.77353300 1.16038700

H -3.03459100 -0.66505200 1.48997200

H -1.58274300 -1.65694200 1.56049400

C -4.45634300 -2.26648900 -1.07255000

H -4.11035900 -0.14673200 -0.80666000

H -3.36732800 -0.80851000 -2.25834300

C -4.71566500 -2.61991200 0.39137000

H -3.60137700 -2.97793300 2.21789500

H -2.85712900 -3.64422800 0.77320500

H -5.40203400 -2.10966400 -1.60134100

H -3.96170400 -3.11345600 -1.56723300

H -5.30686400 -3.53916200 0.46101300

H -5.31391600 -1.82464600 0.85466500

C -3.75290600 3.58724100 1.33145200

H -4.14109000 3.45181400 2.34367900

H -4.55464800 3.90495600 0.65742700

H -2.95606100 4.33962100 1.33214100

O -3.24657200 2.33056200 0.94078700

O -2.76861700 2.43695800 -0.35857500

ethyl 4-(cyclohexylamino)benzoate **(13)**

Reactant Free Energy = -789.058113

0 1

C 4.27348700 -0.71527300 0.81116600

C 5.36141700 0.34844000 0.95729200

C 5.59474900 1.10248100 -0.35152000

C 4.29253900 1.69659800 -0.88742900

C 3.21451500 0.62359700 -1.04013900

C 2.96994300 -0.11676900 0.28179600

H 6.34195000 1.88974500 -0.20605500

H 5.06389200 1.06099400 1.73787000

H 6.29060300 -0.11685500 1.30203000

H 4.61751300 -1.49099100 0.11121900

H 4.08337300 -1.21056200 1.76887700

H 3.93697200 2.47386000 -0.19744200

H 4.46502500 2.19306200 -1.84794400

H 2.27791200 1.05973800 -1.40158200

H 3.52899600 -0.11234100 -1.79245100

H 2.61622400 0.60611000 1.02872700

H 6.00956100 0.41194200 -1.09792900

N 1.96911400 -1.16004900 0.17691200

H 2.30316600 -2.05354500 -0.14964000

C 0.61584700 -0.96080200 0.07860700

C 0.01121900 0.29082600 0.30870700

C -0.22221100 -2.05497200 -0.22858300

C -1.36704500 0.42930300 0.24484600

C -1.59241400 -1.90427700 -0.29189000

H 0.22329400 -3.02938600 -0.40867100

C -2.19154500 -0.65929500 -0.05509800

H -1.81431500 1.39945200 0.42678900

H -2.22963900 -2.74980600 -0.52642800

C -3.65938800 -0.55724200 -0.13527700

O -4.40445100 -1.48339900 -0.39307900

O -4.11041900 0.68995300 0.11077800

C -5.53277300 0.86221600 0.05199400

H -5.88325900 0.57232000 -0.94244000

H -6.00056800 0.18797800 0.77470000

C -5.83063500 2.31169200 0.35608400

H -6.90992900 2.48197000 0.32178900

H -5.47238200 2.58514400 1.35139800

H -5.35671700 2.97132400 -0.37474400

H 0.61726900 1.15862600 0.53862500

Transition State Free Energy = -979.185710

0 2

C -2.48097500 -1.78864700 1.11125100

C -3.33708100 -3.00975200 0.77571400

C -4.66121800 -2.60701400 0.12871800

C -4.42676700 -1.72878900 -1.09942500

C -3.57674700 -0.50595700 -0.76121300

C -2.24194900 -0.91633900 -0.13593700

H -5.23636500 -3.49836000 -0.14320000

H -2.78391000 -3.66718700 0.09147100

H -3.51602200 -3.59117300 1.68630500

H -2.98294500 -1.17144500 1.86580700

H -1.51976100 -2.09193400 1.53905500

H -3.92585400 -2.31978600 -1.87810700

H -5.38242900 -1.40568000 -1.52507200

H -3.38556300 0.09291400 -1.65715600

H -4.11103800 0.14078900 -0.05544200

H -1.69709600 -1.51738000 -0.87819200

H -5.26600600 -2.05322800 0.85868900

N -1.47341200 0.25870000 0.21843600

H -1.97824700 1.34884400 0.12070600

C -0.12119100 0.27982300 0.18061100

C 0.52564500 1.51501600 0.47641500

C 0.69406100 -0.84665600 -0.11695500

C 1.89915300 1.61629600 0.43988800

C 2.07031000 -0.73259400 -0.12721700

H 0.24278700 -1.80728700 -0.33042600

C 2.69141400 0.49729000 0.14039200

H 2.39184300 2.55867400 0.65014300

H 2.68283500 -1.59795000 -0.34996400

C 4.16277700 0.66707600 0.11922500

O 4.81203600 -0.47102500 -0.17935700

O 4.73085700 1.71679700 0.34229000

C -4.48441700 3.23404800 -0.49844000

H -5.42542400 3.35823200 0.04221400

H -4.63288400 2.59644600 -1.37672000

H -4.10021800 4.20840800 -0.81526300

O -3.59621800 2.62821200 0.41512800

O -2.36168800 2.49303800 -0.21488200

C 6.24542400 -0.37844900 -0.22969900

H 6.60638300 -0.00697800 0.73293800

H 6.52096000 0.35576700 -0.99154800

C 6.78236000 -1.75364500 -0.54617800

H 6.40794800 -2.11045000 -1.50852000

H 7.87369700 -1.71916200 -0.59563100

H 6.49776700 -2.47338500 0.22485100

H -0.09125800 2.37399100 0.71566600

ethyl 3-(benzylamino)-4-(cyclohexylamino)benzoate **(14)**

Reactant Free Energy = -1114.598567

0 1

C 0.17693400 2.22960800 0.17675400

C 1.33403500 1.55714400 -0.20941800

C 1.24758000 0.22825100 -0.64967300

C 0.02862600 -0.42822800 -0.70026500

C -1.14734600 0.24330200 -0.26169100

C -1.05072700 1.57602800 0.15051300

H 0.23534700 3.25865400 0.50870900

H 2.16410500 -0.26638600 -0.94580200

H -1.93764700 2.10728900 0.47293300

N -0.12640400 -1.75642300 -1.13130300

H -0.92975600 -1.84802400 -1.74122900

N -2.33369400 -0.47217600 -0.29441800

H -2.18731500 -1.45689700 -0.10833200

C 2.66753700 2.19143800 -0.19871600

O 3.69588400 1.64644000 -0.55107200

O 2.63727000 3.46405800 0.24510600

C 3.89923300 4.14362500 0.27650500

H 4.58519900 3.58538200 0.91964000

H 4.32378900 4.14774200 -0.73124400

C 3.65254600 5.54203200 0.79204600

H 3.22760200 5.51958000 1.79837500

H 4.59570700 6.09343400 0.83064600

H 2.96489100 6.08496000 0.13921000

C -3.58331500 0.05146900 0.23501200

C -4.28782100 0.95737200 -0.78074000

C -4.49694500 -1.11128600 0.62200400

H -3.38471800 0.63580200 1.14930800

C -5.62066100 1.47037800 -0.23722800

H -4.45585200 0.37142200 -1.69330500

H -3.63769600 1.79088600 -1.06298900

C -5.84003200 -0.61750000 1.15923400

H -4.66263700 -1.73723800 -0.26577200

H -3.99892800 -1.73869200 1.37110500

C -6.53500500 0.31512200 0.16739500

H -6.11081200 2.10072300 -0.98626100

H -5.43828100 2.11221700 0.63538200

H -6.48129000 -1.47295900 1.39525900

H -5.67479100 -0.08302900 2.10391800

H -7.46642700 0.69740200 0.59828100

H -6.81510500 -0.25371600 -0.72919800

C 1.02077800 -2.49097300 -1.62964500

H 1.60482200 -1.90502900 -2.35599300

H 0.62420000 -3.35345100 -2.17635100

C 1.94456300 -2.99454500 -0.54030100

C 3.30788400 -3.14726300 -0.80018900

C 1.45303100 -3.35909400 0.71424600

C 4.16388000 -3.66460500 0.16907400

H 3.70737600 -2.84856800 -1.76572100

C 2.30712000 -3.87215400 1.68699600

H 0.39723900 -3.22424800 0.92400100

C 3.66506100 -4.03002500 1.41706500

H 5.22185900 -3.77005400 -0.04737300

H 1.91178900 -4.14574300 2.66005400

H 4.33103300 -4.42583900 2.17665300

Transition State Free Energy = -1304.740901

0 2

C -0.93512800 2.43013000 -0.20326800

C -1.91368200 1.53437200 0.26190400

C -1.56927800 0.21156700 0.50647900

C -0.26869600 -0.26524700 0.29072100

C 0.74184800 0.65710300 -0.17886800

C 0.35742100 1.99880700 -0.40989600

H -1.19913100 3.46318600 -0.39136300

H -2.34905700 -0.45832900 0.84464600

H 1.09808800 2.70867000 -0.75425800

N 0.05170800 -1.57972600 0.47024500

H 1.04280400 -1.82787800 0.63177900

N 2.00355200 0.22176800 -0.39380600

H 2.27383500 -0.71637600 -0.06722100

C -3.31187200 1.94178100 0.51661600

O -4.17656400 1.20622300 0.95030100

O -3.53591000 3.23570600 0.21955300

C -4.87224500 3.70661400 0.44821400

H -5.56402200 3.10234400 -0.14488700

H -5.12281800 3.55547700 1.50176200

C -4.91646100 5.16518500 0.05863900

H -4.66109000 5.29770700 -0.99532700

H -5.92349700 5.55893500 0.21894900

H -4.21948100 5.75368000 0.65997200

C 3.12149400 1.06778900 -0.77542300

C 3.71041800 1.82520600 0.42365600

C 4.19538100 0.21382300 -1.45214100

H 2.76200100 1.79628000 -1.51351500

C 4.90618200 2.67961500 0.00377400

H 4.02099300 1.08560600 1.17153800

H 2.93691800 2.44302900 0.89132300

C 5.39481600 1.06429500 -1.86924400

H 4.51816900 -0.56718600 -0.75271700

H 3.76196000 -0.29480800 -2.31933400

C 5.97805500 1.83746500 -0.68689400

H 5.32393400 3.18830200 0.87889400

H 4.56809100 3.46938600 -0.68122400

H 6.15955400 0.42478400 -2.32200000

H 5.08446000 1.77402900 -2.64811400

H 6.80502800 2.47338100 -1.02052100

H 6.39942400 1.12710100 0.03636300

C -0.88791700 -2.54090500 1.00812700

H -1.39246600 -2.15104200 1.90267000

H -0.28911700 -3.39654100 1.33452800

C -1.92537600 -3.01444100 0.00861500

C -3.22733200 -3.29165900 0.42769100

C -1.58570900 -3.22480100 -1.32954700

C -4.17434900 -3.77802700 -0.47111400

H -3.50889700 -3.11591100 1.46249800

C -2.53109100 -3.70766600 -2.22975600

H -0.57809500 -2.99803500 -1.66318100

C -3.82820900 -3.98811200 -1.80332100

H -5.18456400 -3.98101800 -0.13129500

H -2.25488500 -3.86375400 -3.26758000

H -4.56557200 -4.36088700 -2.50647600

C 3.69150600 -2.97059300 2.63713100

H 4.42440700 -2.63601000 3.37411800

H 4.06220900 -3.85042100 2.10329000

H 2.74232600 -3.20832200 3.12840600

O 3.52287800 -1.88846700 1.74242100

O 2.63247500 -2.25389700 0.76405700

ethyl 3-((3-chlorobenzyl)amino)-4-(cyclohexylamino)benzoate **(15)**

Reactant Free Energy = -1574.214700

0 1

C -0.97762400 2.37873800 0.17062500

C 0.32472800 2.04598800 -0.19378800

C 0.61519200 0.73706800 -0.60478700

C -0.37356700 -0.23259900 -0.64628100

C -1.69469400 0.09460900 -0.23031800

C -1.97583400 1.40989000 0.15150200

H -1.21031300 3.38999100 0.48006400

H 1.63639100 0.51225200 -0.88686700

H -2.97924600 1.68083400 0.45557300

N -0.14869600 -1.56026700 -1.04764400

H -0.89837000 -1.89201300 -1.64212000

N -2.63701000 -0.92211800 -0.25584500

H -2.23299700 -1.82195200 -0.02674300

C 1.43188600 3.02406900 -0.18889300

O 2.57384600 2.77497300 -0.52337500

O 1.04767700 4.24652400 0.22778000

C 2.07493700 5.24703900 0.25427000

H 2.88603600 4.90296800 0.90180300

H 2.48432600 5.35947900 -0.75350400

C 1.45290100 6.52798500 0.75826900

H 1.04752200 6.39741000 1.76443000

H 2.20904800 7.31667000 0.79321800

H 0.64489300 6.85741500 0.10065400

C -3.99763600 -0.74447900 0.22862600

C -4.88878600 -0.09419300 -0.83479700

C -4.57188600 -2.10050400 0.63832000

H -3.99352200 -0.10233900 1.12553300

C -6.32804900 0.05051800 -0.34240100

H -4.86034100 -0.72872300 -1.72967000

H -4.48100800 0.87715000 -1.13040100

C -6.01512200 -1.97586900 1.12604500

H -4.53324600 -2.77477500 -0.22852700

H -3.94618800 -2.54544000 1.42156400

C -6.90510300 -1.29850000 0.08439900

H -6.94654000 0.49932600 -1.12635000

H -6.35685000 0.74412600 0.50891400

H -6.40689000 -2.96658200 1.37835500

H -6.03237300 -1.38950400 2.05405400

H -7.91901600 -1.17351200 0.47882400

H -6.98957200 -1.94853200 -0.79663600

C 1.15434300 -1.95304300 -1.54334600

H 1.55270500 -1.23589100 -2.27752700

H 1.01518000 -2.89834000 -2.07866000

C 2.18421500 -2.17119800 -0.45339900

C 3.53997400 -2.06312200 -0.76841500

C 1.81156900 -2.52340800 0.84361600

C 4.49804200 -2.31993200 0.20562700

H 3.85368300 -1.77354600 -1.76601400

C 2.78245500 -2.76922000 1.81050800

H 0.75780600 -2.59123800 1.08825100

C 4.13628000 -2.67347500 1.50133500

H 2.48546200 -3.03617200 2.81932500

H 4.89820100 -2.86097900 2.24822800

Cl 6.18668500 -2.18656000 -0.20511100

Transition State Free Energy = -1764.357105

0 2

C -0.25717600 2.78804400 0.20050400

C 0.93523300 2.20722500 -0.26846600

C 0.98129800 0.84228600 -0.51427900

C -0.13036000 0.01452900 -0.29764800

C -1.36333800 0.61301200 0.16928600

C -1.37367200 2.00835500 0.40692300

H -0.29589100 3.85326200 0.39050200

H 1.91970300 0.42377700 -0.85507700

H -2.28553100 2.47728800 0.75254500

N -0.06262800 -1.33722400 -0.46556300

H -0.94464600 -1.86220800 -0.63167100

N -2.45070700 -0.16003000 0.37535200

H -2.44403800 -1.13675000 0.04243100

C 2.16108600 2.99530800 -0.52324200

O 3.19914000 2.53235200 -0.95207300

O 2.00680300 4.29973200 -0.23191500

C 3.15580800 5.13044600 -0.45965300

H 3.98686500 4.75199200 0.14152100

H 3.44523800 5.04864600 -1.51080600

C 2.78055200 6.54390600 -0.08280200

H 2.49242400 6.60646300 0.96918600

H 3.63477800 7.20664100 -0.24361300

H 1.94831500 6.90493100 -0.69166300

C -3.75893200 0.33192400 0.77438100

C -4.53959700 0.92350500 -0.40854900

C -4.54761400 -0.80620400 1.42513500

H -3.61279200 1.11384800 1.53120600

C -5.92640300 1.39570600 0.02712000

H -4.63169600 0.14603200 -1.17663800

H -3.97248100 1.74541200 -0.85738400

C -5.93805900 -0.34009200 1.85493900

H -4.63627200 -1.62891500 0.70497300

H -3.98712000 -1.19286600 2.28236700

C -6.71605700 0.26669100 0.68792000

H -6.47170000 1.79020400 -0.83655900

H -5.82354400 2.22908400 0.73576100

H -6.49047300 -1.18126900 2.28607200

H -5.84109300 0.40795200 2.65355400

H -7.68925500 0.63346800 1.03111600

H -6.91873800 -0.51433700 -0.05646600

C 1.11592900 -1.99530100 -0.98161600

H 1.50763200 -1.48366800 -1.87123800

H 0.79062400 -2.98709500 -1.30994100

C 2.22845900 -2.15911200 0.03763500

C 3.54711200 -2.27195500 -0.40528000

C 1.95457000 -2.24980800 1.40241900

C 4.56752600 -2.48289900 0.51553000

H 3.78562900 -2.19020600 -1.46069000

C 2.98806400 -2.45421100 2.31200400

H 0.93076000 -2.15170800 1.74610100

C 4.30525300 -2.57552400 1.87830000

H 2.76901700 -2.51970900 3.37251500

H 5.11566200 -2.73305500 2.57975300

C -3.14887500 -3.65899400 -2.66148200

H -3.90871400 -3.50574700 -3.43073000

H -3.34333300 -4.58595700 -2.11391800

H -2.15382100 -3.70122400 -3.11687700

O -3.24679400 -2.54633300 -1.79606600

O -2.32936300 -2.69371500 -0.77918700

Cl 6.20910100 -2.62475400 -0.05208200
